# Influencing factors and solution strategies of chimeric antigen receptor T-cell therapy (CAR–T) cell immunotherapy

**DOI:** 10.32604/or.2024.048564

**Published:** 2024-08-23

**Authors:** ZHENGYI WANG, LIANG ZHOU, XIAOYING WU

**Affiliations:** 1 Department of Institute of Laboratory Animal Sciences, Sichuan Provincial People’s Hospital, University of Electronic Science and Technology of China, Chengdu, China; 2 Ministry of Education and Training, Chengdu Second People’s Hospital, Chengdu, China

**Keywords:** Chimeric antigen receptor T-cell therapy (CAR–T), Tumor targeting therapy, Influencing factor, Solution strategies

## Abstract

Chimeric antigen receptor T-cesll therapy (CAR–T) has achieved groundbreaking advancements in clinical application, ushering in a new era for innovative cancer treatment. However, the challenges associated with implementing this novel targeted cell therapy are increasingly significant. Particularly in the clinical management of solid tumors, obstacles such as the immunosuppressive effects of the tumor microenvironment, limited local tumor infiltration capability of CAR–T cells, heterogeneity of tumor targeting antigens, uncertainties surrounding CAR–T quality, control, and clinical adverse reactions have contributed to increased drug resistance and decreased compliance in tumor therapy. These factors have significantly impeded the widespread adoption and utilization of this therapeutic approach. In this paper, we comprehensively analyze recent preclinical and clinical reports on CAR–T therapy while summarizing crucial factors influencing its efficacy. Furthermore, we aim to identify existing solution strategies and explore their current research status. Through this review article, our objective is to broaden perspectives for further exploration into CAR–T therapy strategies and their clinical applications.

## Introduction

Tumor immunotherapy is a therapeutic method to control and destroy tumors by restarting and maintaining the tumor immune cycle and restoring the body’s normal anti-tumor immune response. In recent years, researchers have made significant innovative achievements in the field of tumor immunotherapy based on the knowledge of biology, oncology and immunology. In the past decade, tumor immunotherapy technology has made significant advancements, particularly in the field of hematologic malignancies, leading to remarkable clinical outcomes and unprecedented improvements in treatment efficacy. The utilization of immunotherapy presents a promising strategy to elicit a more robust immune response in patients with advanced malignancies, as compared to conventional chemotherapy.

Several immunotherapies have been developed and implemented in clinical practice, including: (1) monoclonal antibodies and their enhanced formulations, such as antibody-drug conjugates (ADCs) and bi-specific T-cell engagers (BiTE); (2) immunomodulatory agents aimed at augmenting endogenous antitumor activity, such as immune checkpoint inhibitors (ICIs); (3) adoptive cellular immunotherapy (ACT), which encompasses allogeneic stem cell transplantation (ASCT) and chimeric antigen receptor T-cell therapy (CAR–T), has garnered significant attention [1]. In the early stages of ACT, immunocompetent cells were extracted from cancer patients and utilized in adoptive therapy. These cells were characterized for their function *in vitro* before being transfused back into the patient to stimulate their immune system and eradicate tumor cells. The application of immune cells has been restricted in clinical settings due to various challenges, including the limited expansion rate [2]. Subsequently, CAR–T immunotherapy was developed to recognize tumor cells based on modified T-cell antigen receptors. Compared with conventional chemotherapy, CAR–T therapy has significantly improved efficacy in patients with acute lymphoblastic leukemia and is considered the most promising adoptive immunotherapy for cancer. Clinical ACT therapy typically involves two approaches: (1) the retrieval of tumor-infiltrating lymphocytes from the patient’s primary tumor tissue, their subsequent *in vitro* expansion, and autologous reinfusion; (2) For the generation of circulating T lymphocytes, a gene modification approach was employed to engineer T cells expressing specific tumor antigens. The generation of monoclonal T cells with predetermined antigen specificity has been achieved through two genetic modification approaches: transfer of T-cell receptor (TCR) genes and transfer of chimeric antigen receptor (CAR) genes.

The CAR cells are genetically modified to express antigen-specific, non-MHC-restricted receptors, known as synthetic modular peptides. These peptides bind to target antigens expressed on neighboring cells’ surfaces and deliver signals for cell activation. This antigen receptor combines the antibody’s specificity with its signaling capacity to activate the receptor, thereby facilitating targeted migration towards the tumor site and augmenting its tumor-specificity. The chimeric receptor exhibits selective and efficient recognition of tumor-associated antigens (TAAs) expressed by tumor cells, unaffected by the escape mechanism involving loss or down-regulation of major histocompatibility molecules. Consequently, this enhances the efficacy of tumor treatment. The receptor comprises an extracellular antigen recognition region, typically derived from a single-chain variable fragment (scFv) of a monoclonal antibody, which is fused to a hinge, a transmembrane domain, an intracellular signaling domain, and/or a costimulatory molecule [3]. CAR–T cell therapy has demonstrated remarkable efficacy in various hematologic malignancies [4,5]. However, these studies also highlight significant clinical challenges, such as the emergence of treatment resistance in a subset of patients, the difficulty in transitioning to solid tumors, and the potential for treatment-related toxicity [6]. The field of CAR–T therapy encounters numerous challenges in the context of solid tumors, and the determinants of its success or failure may exhibit a multimodal nature. In contrast to hematological malignancies, the identification of an optimal single-target antigen in solid tumors poses a greater challenge. While the overexpression of TAAs is frequently observed in tumors, their expression at physiological levels is also detected in normal non-tumor tissues. The proteins commonly targeted in solid tumors include epidermal growth factor receptor (EGFR), carcinoembryonic antigen (CEA), epidermal growth factor receptor 2 (ERBB2), prostate-specific membrane antigen (PSMA), and mesothelin. The lack of tumor antigen specificity in CAR–T cells poses a clinical challenge for conventional cancer treatment due to the increased risk of non-tumor toxicity in normal tissues. Challenges also arise from inadequate knowledge of appropriate tumor-specific antigens (TSAs)/TAAs, the heterogeneity of tumor antigens, difficulties in CAR–T cell infiltration into the tumor site, negative impacts of the tumor microenvironment on CAR–T cells, as well as issues related to CAR–T cell proliferation and endurance. This review primarily discusses recent research findings aimed at enhancing the therapeutic efficacy and minimizing adverse reactions of CAR–T cells in tumor immunotherapy. It comprehensively evaluates the merits and drawbacks of various pre- and post-clinical application methods, while also identifying future directions for development to identify treatment concepts and approaches that are more suitable for clinical implementation.

## Factors Influencing the Efficacy of CAR–T Cell Therapy in the Context of Solid Tumor Treatment

In the treatment of hematologic malignancies, infused CAR–T cells efficiently bind to tumor cells in the vascular lumen, enabling precise targeting of molecular markers. However, the application of CAR–T therapy for solid tumors presents increasing challenges due to intricate interactions among tumor microenvironment, immune response, and stromal cell communication. Several unfavorable factors significantly impact the efficacy of solid tumor CAR–T cell therapy.

### The infiltration of CAR–T cells into the tumor microenvironment poses a significant challenge

Limited infiltration of immune cells has significantly impeded the therapeutic efficacy of CAR–T cell therapy in solid tumors. Unlike circulating tumor cells, which can spread through the blood and lymphatic systems, solid tumors are surrounded by their own tissue barriers. Within solid tumors, there is a high interstitial fluid pressure (IFP) at the core of the tumor, causing fluids to flow towards less dense regions outside of the tumor [7]. The tumor core exhibits reduced capillary perfusion compared to the periphery, resulting in limited access of cells near the core to immune cells, nutrients, and oxygen from extracellular circulation [8]. Furthermore, the migration of these redirected effector cells across the vascular endothelium into the tumor tissue may be impeded [9]. Tumor angiogenic conditions can induce the formation of dysfunctional blood vessels and nonreactive endothelial cells, resulting in a nonadherent inner layer of endothelium that further hinders the effective infiltration of leukocytes into the tumor [10]. Moreover, the chemokine axis plays a crucial role in regulating T cell migration. The dysregulation of chemokines by tumor cells or tumor-associated cells can result in inadequate recruitment of CAR–T cells within the tumor microenvironment [11]. For instance, tumors secrete chemokines such as CCL2, CCL3, CCL5, CCL17 and CCL22 which are the primary chemokines for immunosuppressive cells to migrate towards tumors. However, these chemokines do not facilitate the recruitment of cytotoxic CD8+, CD4+T cells or CAR–T cells. This barrier impedes the efficient infiltration of intravenously injected CAR–T cells into tumor sites resulting in tumor protection.

### Impact of the tumor immunosuppressive microenvironment on CAR–T cell activity

Tumor microenvironment (TME) is a complex system comprising diverse cell types (e.g., immune cells, vascular endothelial cells, and fibroblasts), extracellular matrix components (e.g., collagen), and secreted factors (e.g., cytokines). These elements are critical determinants of anti-tumor immunity as they can impede the infiltration, activation, and effector function of tumor-specific T cells, thereby posing a significant challenge to effective immunotherapy. Tumor growth is influenced by TME, wherein diverse immune cell populations and non-malignant cells, such as fibroblasts, engage in intricate interactions with tumor cells to orchestrate immune tolerance mechanisms, thereby impacting the clinical efficacy of immunotherapy. Among them, bone marrow-derived suppressor cells (MDSCs), regulatory T cells (Tregs), and tumor-associated macrophages (TAMs) represent the principal immunosuppressive cell populations. Through their interactions within the TME, they exert regulatory control over tumorigenesis and tumor progression at various levels.

#### The impact of TAM on the functionality of CAR–T cells

Macrophages are a heterogeneous population of immune cells derived from the myeloid lineage, primarily involved in phagocytic functions during immune responses and tissue remodeling. The activation state of macrophages is traditionally categorized into M1 (classically activated) and M2 (alternatively activated) macrophages; however, the observed phenotypic plasticity cannot be simply dichotomized, but rather represents a spectrum ranging from pro-inflammatory to anti-inflammatory responses. The former is associated with antitumor and anti-infective effects, while the latter contributes to wound healing and tumor support effects. In the TME, macrophages are commonly referred to as TAMs. TAMs exhibit a highly heterogeneous and plastic cellular composition, capable of both promoting tumor progression (M2 phenotype) and augmenting anti-tumor immunity (M1 phenotype) [12]. Under the recruitment of chemokines, including CCL2, CCL20, CXCL12, and CSF-1 within the tumor microenvironment, macrophages in close proximity to solid tumors undergo rapid reprogramming towards M2-like phenotypes driven by local hypoxia [13]. Polarized TAMs facilitate tumor progression through the upregulation of interleukin-6 (IL-6), vascular endothelial growth factor (VEGF), inducible nitric oxide synthase (iNOS), arginase, and indoleamine 2,3-dioxygenase (IDO)-1/2. The secretion of CCL2 recruits and activates other immunosuppressive cell subsets including Tregs and MDSCs, while also engaging inhibitory coreceptors PD-1 and CTLA-4 to induce checkpoint blockade [14]. Additionally, direct inhibition of effector cells such as natural killer cells (NK cells) and cytotoxic T lymphocytes promotes immune suppression within the tumor microenvironment. Up-regulation of HIF1α/2α can induce the upregulation of pro-angiogenic factors, thereby facilitating tumor neovascularization [15]. It stimulates extracellular matrix remodeling and facilitates tumor metastasis through the secretion of factors such as platelet-derived growth factor (PDGF), transforming growth factor-beta (TGF-β), matrix metalloproteinase 2 (MMP2) and MMP9 [16]. For instance, the promotion of tumor release from the primary site and establishment in the secondary site can be facilitated by increasing vascular permeability [17].

Numerous studies conducted on TAMs across various malignancies have consistently demonstrated a strong correlation between elevated TAM levels and unfavorable prognostic outcomes, particularly in immuno-cold tumors characterized by extensive infiltration of TAMs with dismal prognosis [18,19]. Sanchez et al. devised a strategy to deplete TAMs in a murine solid tumor model by employing CAR–T cells that specifically target the F4/80 macrophage marker. *In vitro* and *in vivo* experiments demonstrated the potent cytotoxicity of F4.CAR–T cells against macrophages. Furthermore, infiltration of CAR–T cells into the tumor microenvironment resulted in suppressed tumor growth and significantly prolonged survival in mice [20].

In-depth investigations have revealed that the TAM phenotype classification in progressive tumors is predominantly dominated by M2 type. In tumor therapy, targeting M2 or inducing a shift from M2 to M1 type and increasing the intratumoral ratio of M1/M2 can enhance the disease prognosis. In a phase I trial evaluating anti-CD19 CAR–T cells for patients with relapsed/refractory B-cell non-Hodgkin’s lymphoma (B-NHL) (NCT03355859), researchers assessed the expression of CD68, a general marker of macrophage lineage, and CD163, a marker associated with M2 alternative activation and anti-inflammatory macrophages [21]. The poor prognosis was significantly associated with an increased infiltration of CD68^+^ and CD163^+^ macrophages. The clinical findings should validate their previous investigation, which demonstrated that co-culturing M2 macrophages significantly attenuated the proliferation of CD4+ and CD8+ T cells in comparison to T cell culture alone. Some studies have reported that the subset of TAMs expressing folate receptor β (FRβ) exhibits M2-like immunosuppressive properties [12]. CAR–T cell-mediated targeted elimination of FRβ+ TAMs within the TME leads to an enhanced recruitment of proinflammatory monocytes, infiltration of endogenous CD8+ T cells specific to the tumor, resulting in a decelerated progression of tumors and prolonged survival in a syngeneic mouse model. Preconditioning the TME with FRβ-specific CAR–T cells enhanced the therapeutic efficacy of anti-mesothelin CAR–T cells, whereas co-administration of both CAR products failed to achieve comparable outcomes. These findings highlight the significance of preemptively eliminating M2 phenotype cells to augment the efficacy of tumor-targeting CAR therapy. Furthermore, Yamaguchi et al. observed that the activity of CAR–T cells was suppressed in the presence of M2 macrophages rather than M1 macrophages. Additionally, they found a correlation between infiltration of CD163^+^ M2 macrophages and tumor progression as well as an unfavorable response to immunotherapy [22]. IFN-γ signaling induces the depletion of CD163^+^ M2 macrophages, thereby enhancing the antitumor efficacy of CAR–T cells. Hao et al. utilized single-cell RNA sequencing (scRNA-seq) and mass spectrometry techniques to identify that APOC1 exhibited significantly elevated expression levels in TAMs of hepatocellular carcinoma (HCC) tissues compared to normal tissues [23]. Inhibition of APOC1 can reverse the M2-to-M1 transformation of liver cancer TAMs through the ferroptosis pathway. Tumors in APOC1^−/−^ C57BL/6 mice exhibited consistent attenuation compared to wild-type (WT) mice. The relative proportions of M2 macrophages, B cells, and CD4^+^ T cells in the APOC1^−/−^ group showed a downward trend compared with the WT group, while the relative proportions of CD8^+^ T cells, M1 macrophages, and NK cells showed an upward trend. These findings provide a novel strategy for enhancing immunotherapy efficacy.

The presence of M2 isoforms in the tumor microenvironment TAM has been shown to impact the efficacy of immunotherapy, and successful activation of CAR–T activity necessitates the elimination or transformation of M2 isoforms.

To evaluate the impact of immunosuppressive TAMs and MDSCs on CAR–T cell efficacy, Luo et al. employed a folate-targeted Toll-like receptor 7 agonist (FA-TLR7-1A) to selectively reinvigorate TAMs and MDSCs from an immunosuppressive state to a proinflammatory phenotype, while leaving the characteristics of other immune cells unchanged [24]. The inclusion of FA-TLR7-1A significantly augmented the efficacy of standard CAR–T cell therapy against 4T1 solid tumors in immunocompetent mice. Moreover, the combination of FA-TLR7-1A and CAR–T cell therapy not only reprogrammed TAMs and MDSCs from an M2-like anti-inflammatory phenotype to an M1-like pro-inflammatory phenotype but also facilitated enhanced infiltration and activation of both CAR–T cells and endogenous T cells within solid tumors, without any notable adverse effects observed. Therefore, M2 macrophages in the TME represent a crucial factor influencing the efficacy of CAR–T therapy. Pre-clearance or polarization of these cells towards an M1 phenotype could significantly enhance clinical outcomes. However, to date, no clinical studies have investigated targeted CAR therapy specifically directed against M2 macrophages or combined with CAR–T targeting other TAAs. The potential benefits and adverse effects of this strategy remain unknown and warrant further investigation.

#### To elucidate the impact of MDSCs on the functionality of CAR–T cells

The accumulation of MDSCs in tumor hosts is a hallmark of malignancy-related inflammation and a major contributor to the induction of T-cell suppression within tumors. Depletion of MDSC subsets through anti-GR-1 antibody treatment has been shown to induce CD8^+^ T cell-mediated antitumor effects in mouse models [25]. MDSCs are a heterogeneous population of myeloid cell lineages, encompassing myeloid progenitors such as immature macrophages, granulocytes, and dendritic cells (DCs), which are diverse immune cells of the myeloid lineage that participate in multiple immune processes. The subsets of MDSCs can be primarily classified into two categories: polymorphonuclear MDSCs (PMN-MDSCs) and mononuclear MDSCs (M-MDSCs). In the majority of malignant tumors, M-MDSCs and PMN-MDSCs constitute approximately 20% and 80% of the total MDSC population, respectively [26]. In humans, due to the lack of specific markers for MDSCs, only combinations of common immune markers can be utilized for their identification. Human M-MDSCs were characterized as CD33^+^CD11b^+^CD14^+^CD15^−^HLA-DR^−/low^, while human PMN-MDSCs were identified as CD33^dim^CD11b^+^CD15^+^CD14^−^HLA-DR^−/low^. Furthermore, a third subset known as early MDSCs (eMDSCs), which lack myeloid markers (CD14, CD15, and CD66b) but express the surface marker combination of CD33^+^CD11b^+^ and HLA-DR^−/low^ has been described in humans [27]. The inhibition of T cell activation represents a crucial characteristic of MDSCs. MDSCs are responsive to various metabolic factors, cytokines, and growth factors within the TME, leading to an upregulation in the expression of immunosuppressive factors that ultimately impede T cell function [28]. The inhibitory effect of MDSCs on CAR–T cells primarily correlates with the following factors.

##### MDSCs exert an impact on nutrient metabolism, thereby disrupting the supply of essential amino acids to CAR–T cells

CAR–T cells are genetically modified T cells engineered to express the CAR structure. Their metabolic and nutritional requirements closely resemble those of native T cells, exhibiting substantial similarity.

##### Aberrant metabolism of arginine

L-arginine (L-Arg) is an indispensable nutrient for T cell survival and serves as a substrate for four metabolic enzymes, which exist in multiple isoforms: nitric oxide synthase (NOS1, NOS2, and NOS3), arginase (arginase I and II, Arg1/Arg2), arginine: glycine aminotransferase (AGAT), and L-arginine decarboxylase (ADC). The enzyme Arg1/Arg2 catalyzes the conversion of L-Arginine to L-ornithine and urea [28]. L-Arg is metabolized by NOS enzymes to produce citrulline and nitric oxide [29]. The involvement of ADC and AGAT in the immune response appears to be relatively limited [30]. Multiple studies have demonstrated the presence of Arg1 and Arg2 in diverse tumor types, with their heightened activity typically associated with advanced disease progression and unfavorable clinical prognosis. This encompasses malignancies affecting the head and neck region [31], neuroblastoma [32], Acute myeloid leukemia (AML) [33]. Patients diagnosed with AML, neuroblastoma, and childhood cancer exhibit significantly reduced levels of arginine in their peripheral blood compared to healthy controls. Furthermore, the expression of Arg1 is observed within the tumor microenvironment of various adult tumor subtypes [34]. Previously, this mechanism was believed to be responsible for maintaining the production of polyamines necessary for facilitating rapid proliferation of tumor cells. However, Raber et al. demonstrated that Arg1 was preferentially expressed in tumors infiltrated by MDSCs, which suppressed T cell immune responses, exerted immunosuppressive effects on tumors, and promoted rapid tumor cell proliferation [35]. Subsequent reports have demonstrated that the low arginine microenvironment hinders the immune response of antigen-specific CAR–T cells by impeding T cell proliferation [32,33]. In the tumor microenvironment, PMN-MDSCs are the primary source of ArgI, while M-MDSCs metabolize L-Arg through NOS2 [35]. Overexpression of Arg1 in MDSCs results in local depletion of L-Arginine within the tissue [36]. The primary mechanism by which MDSCs induce T cell tolerance is through the consumption of extracellular L-Arginine via Arginase I, resulting in a low arginine tumor microenvironment that may impair L-Arginine uptake by T cells and alter their metabolic profile. *In vitro* addition of ArgI inhibitors or their injection into tumor-bearing mice effectively preserved T-cell function and elicited an immune-mediated antitumor response, leading to dose-dependent inhibition of tumor growth. These responses were absent in immunodeficient SCID mice bearing tumors, strongly indicating that the antitumor effect induced by arginase I inhibition is reliant on lymphocyte function [35]. Further investigations have demonstrated that alterations in arginine metabolism can result in reduced levels of L-Arg within T cells [37]. Enhanced levels of L-Arginine induced significant metabolic alterations in activated T cells, including a transition from glycolysis to oxidative phosphorylation (OXPHOS), thereby facilitating the generation of central memory-like cells with augmented viability. One plausible explanation for the transition to OXPHOS is that elevated levels of L-arginine upregulate the serine biosynthesis pathway, which has been demonstrated to fuel the tricarboxylic acid cycle and thereby promote OXPHOS [38]. Proteomic investigation of structural alterations through analysis of knockout T-cell clones revealed three transcriptional regulators (BAZ1B, PSIP1, and TSN) that exhibit sensitivity to L-Arg levels and facilitate the survival of T-cells. The authors propose that the metabolic fitness and viability of T cells, which are crucial for anti-tumor responses, are directly influenced by intracellular L-Arg concentration [37]. T cells are sensitive to extracellular concentrations of arginine, and a low arginine microenvironment can impair the proliferation of CAR–T cells, thereby limiting their efficacy in clinical trials for hematological and solid tumors. Reprogramming cellular metabolism can ameliorate this deficiency. Due to the limited expression of arginine resynthetase—argininosuccinate synthase(ASS) and ornithine transcarbamylase (OTC) in T cells, these cells are more susceptible to a low-arginine microenvironment [39,40]. Reengineering CAR–T cells to express functional ASS or OTC enzymes and to act synergistically with different chimeric antigen receptors can enhance CAR–T cell proliferation without compromising cytotoxicity or inducing exhaustion. Enzyme-modified CAR–T cells exhibit improved efficacy in clearing leukemia or solid tumor burden *in vivo*.

##### Abnormal tryptophan metabolism

L-tryptophan (Trp) is an indispensable amino acid that can only be obtained through dietary intake, and its metabolites play crucial roles in various physiological processes [41]. Over 95% of free tryptophan serves as a substrate for the kynurenine pathway (KP) of tryptophan catabolism [42]. The rate-limiting step in the Kyn pathway involves the enzymatic conversion of tryptophan to N-formylkynurenine (NFK) by indoleamine-2,3-dioxygenase 1 (IDO1), IDO2, and tryptophan-2,3-dioxygenase (TDO). Depletion of tryptophan by these enzymes has profound effects on cellular function and survival [41]. The *TDO2* gene-encoded TDO has long been acknowledged as the principal hepatic enzyme responsible for catalyzing dietary tryptophan degradation. The catalytic activity of TDO is equivalent to that of IDO1, and the activation of TDO exerts an impact on the immune response by suppressing T cell proliferation, restricting tumor immune infiltration, and impeding antitumor immune responses. IDO1 is an inducible enzyme that is widely expressed and encoded by the *IDO1* gene, which belongs to the interferon-activating gene family. Although the IDO2 enzyme, which is associated with IDO1, may potentially contribute to immune tolerance mediated by IDO1, the precise physiological function of IDO2 and its involvement in diseases related to KP activity remain poorly elucidated. Elevated tryptophan catabolism represents a prevalent hallmark of advanced malignancies [43]. The upregulation of IDO1 is a frequent occurrence in human malignancies. Tryptophan degradation is believed to exert immunosuppressive effects through the generation of tryptophan catabolites, such as kynurenine, which can suppress immune cells [44]. The deficiency of Trp (<1 µM) theoretically results in the aggregation of uncharged tRNA and activation of the general control non-derepressible 2 (GCN2) kinase pathway, leading to dysfunction of T cells and antigen presenting cells (APCs) [45]. However, these changes lack a physiological and pathological basis, and their effects on immunosuppression are more likely due to the metabolites of Trp [46]. IDO-dependent MDSCs are a crucial component in the establishment of an immunosuppressive tumor microenvironment, and there exists a close association between IDO-dependent MDSCs and this environment. It has been observed that subpopulations of human or mouse MDSCs express *IDO1*, with its expression correlating to their immunosuppressive function [47]. Loss of *IDO1* led to a decrease in IL-6, the primary inducer of MDSCs, and overexpression of IL-6 was sufficient to restore the impaired T cell suppressive function of MDSCs and reverse the resistance of IDO1^-/-^ mice to lung metastasis [48]. Previous studies have demonstrated a close correlation between IDO expression in human melanoma tumors and MDSC infiltration. *In vivo* treatment with a selective IDO inhibitor resulted in decreased expression of *IDO1*, reduced numbers of tumor-infiltrating MDSCs and Treg cells, and increased infiltration of CD8^+^ T cells. The reversal of the inhibitory function of IDO led to the elimination of tumor-related immunosuppression. Furthermore, it was found that IDO can recruit and activate MDSCs to coordinate local and systemic immunosuppression [47]. Recently, it has been reported that IDO can induce immunosuppression independently of Trp metabolites. Furthermore, studies have demonstrated that inactive IDO can still decrease the survival time of experimental animals and upregulate the expression of complement factor H (CFH) and its isoform factor H-like protein 1 (FHL-1) in human glioblastoma (GBM). Tumor cell IDO increases CFH and FHL-1 expression regardless of tryptophan metabolism. Elevated intratumoral levels of CFH and FHL-1 are associated with poor survival in glioma patients. Similar to the effect of IDO, GBM cell FHL-1 expression enhances intratumoral Tregs and MDSCs while reducing overall survival in GBM mice [49].

##### MDSCs interact with a diverse array of cytokines to exert inhibitory effects on the function of CAR–T cells

Cytokines are signaling proteins that possess the capacity to significantly modulate CAR–T cell function, either by enhancing or attenuating it. MDSCs have been demonstrated to respond to various cytokines and growth factors present in tumors, leading to up-regulation of known immunosuppressive factors and acting as suppressors of T cell or CAR–T cell function [28].

##### Recruitment of chemokines

In humans, MDSCs are actively recruited to both primary and metastatic tumor sitesin cancer such as glioblastoma [50], urothelial carcinoma [51]. The migration of M-MDSCs to tumors is tightly regulated by chemokines secreted by the tumor microenvironment. Notably, CCL2 and CCL5 have been identified as key chemokines orchestrating this process. *In vitro* cultures of human breast, ovarian, and stomach tumors demonstrate secretion of CCL2, with MDSCs from these patients expressing corresponding CCR2 receptors and exhibiting migration towards these chemokines *in vitro* [52]. The expression of CCL2 increases progressively in humans with colorectal cancer, while depletion of CCL2 in a mouse model of spontaneous colorectal cancer leads to a reduction in colonic MDSC numbers [53]. Elevated expression of CCL15 at the invasive front facilitates recruitment of MDSCs expressing CCR1 in Smad4-deficient colorectal cancer [54]. Neutrophils and PMN-MDSC are primarily recruited by CXC chemokines, specifically CXCL1, CXCL5, CXCL6, CXCL8, and CXCL12. Moreover, accumulating evidence suggests that the CCL2 chemokine response, which primarily attracts monocytes and/or M-MDSCs, also facilitates the recruitment of PMN-MDSCs to tumor sites [53]. In a murine model of HCC, the presence of abundant tissue inhibitors of metalloproteinases results in an upregulation of CXCL12 production, thereby facilitating the recruitment of PMN-MDSC to sites of tumor formation within the premetastatic microenvironment through CXCR4 signaling [55]. The recruitment of PMN-MDSC to human melanoma cells via CXCL8 contributes to the promotion of lung metastasis by facilitating the adhesion of melanoma cells to vascular endothelium in a xenograft model [56]. Refer to Table 1 for an overview of the involvement of chemokines in the recruitment process of MDSCs.

**Table 1:** Recruitment of MDSCS to tumors by chemokines*

Chemokine	Cancer	Leukocyte infiltrate	Reference
CCL2 (MCP-1)	Breast,gastric, and ovarian cancer	MDSC	[52]
	Colorectal cancer	MDSC	[53]
CXCL5	Mammary carcinoma (mouse)	MDSC	[57]
	Non-small cell lung cancer	PMN-MDSC	[58]
	Melanoma (mouse)	PMN-MDSC	[59]
CXCL6 (GCP-2)	Gastrointestinal tumors	PMN-MDSC	[60]
	Tongue aauamous cell caroinoma	PMN-MDSC	[61]
CXCL8 (IL-8)	Head and neck squamous cell carcinoma	PMN-MDSC	[62]
	Gastric carcinoma	PMN-MDSC	[63]
	Bronchoalveolar carcinoma	PMN-MDSC	[64]
	Tongue squamous cell carcinoma	PMN-MDSC	[61]
CCL15 (MIP-5)	Colorectal cancer	PMN-MDSC	[54]
CXCL12 (SDF-1)	Colorectal cancer	M-MDSC	[65]
	Mammary carcinoma (mouse)	MDSC	[57]

Note: “*”: MDSC, the total number of MDSC, does not define a specific subset.

##### Immunosuppressive factors and their interactions

The interaction between MDSCs and various environmental factors within the tumor microenvironment leads to their generation, activation, and subsequent exertion of immunosuppressive functions. For instance, chronic inflammation, including inflammation associated with cancer and chronic infection, can stimulate the release of these cells from the bone marrow [28]. Studies have demonstrated that activation of the sympathetic nervous system (SNS) and adrenergic stress can enhance the generation and differentiation of MDSCs. Stimulation of β-adrenergic receptors, particularly β2-AR, on myeloid cells leads to the mobilization of MDSCs from the bone marrow into the bloodstream [66]. Prolonged exposure to elevated levels of catecholamines also facilitated the further differentiation of MDSC into PMN-MDSC, M-MDSC, and macrophages with enhanced immunosuppressive properties within tumor tissues and secondary lymphoid organs, including the spleen [66]. Furthermore, the restoration of tumor immunity was achieved through pharmacological blockade of both β1-AR and β2-AR using propranolol, a nonselective β-blocker [67]. The activation of β-AR signaling in MDSCs has been demonstrated to induce the activation of a key signaling pathway, which is closely associated with the downstream transcription factor STAT3 [66,67]. Activation of the STAT3 pathway induces the expression of numerous downstream functional proteins, thereby facilitating the acquisition of additional immunosuppressive functions by MDSCs and tumor-associated macrophages. The up-regulated immunosuppressive proteins, including VEGF, IL-4 and IL-5, CCR4, and FoxP3, activated their respective immunosuppressive pathways and thereby induced an immunosuppressive effect [68]. Furthermore, it has been demonstrated that GM-CSF and IL-6 effectively induce the activation of the immunosuppressive program in bone marrow-derived progenitor cells through precise regulation of the C/EBPβ transcription factor [69]. The presence of interleukin-1β (IL-1β) in the TME facilitated the recruitment of MDSCs and TAMs, while also activating STAT1 and STAT3 signaling pathways within MDSCs [70]. Interleukin-4 (IL-4) and interleukin-13 (IL-13) elicit activation of the STAT6 pathway in MDSCs. Activation of this signaling cascade induces an immunosuppressive phenotype by upregulating genes such as *ARG1* and *NOX2*, thereby promoting the production of reactive oxygen species (ROS) and reactive nitrogen species (RNS) [71]. Tumor necrosis factor-α (TNF-α) in the tumor microenvironment also exerts a significant impact on MDSCs. TNF-α can impede the differentiation of MDSCs into mature cells, while simultaneously augmenting their inhibitory activity during chronic inflammation, resulting in a dual effect [72].

Mature and activated MDSCs can autonomously or locally produce additional immunosuppressive cytokines, such as IL-10 and TGF-β, thereby influencing the TME. This autocrine or paracrine signaling can induce Treg activation, promote their differentiation and local accumulation, ultimately impacting the functionality of CAR–T and NK cells within the TME [28,36]. Moreover, MDSCs can secrete substantial levels of CCR5 ligands, including CCL3, CCL4, and CCL5, which attract a considerable number of tumor-infiltrating peripheral Tregs expressing CCR5. This phenomenon contributes to the establishment of an immunosuppressive microenvironment within the tumor and impairs the anti-tumor functionality of both conventional T cells and CAR–T cells [73]. In terms of intercellular communication, MDSCs frequently exhibit an upregulation of programmed cell death ligand 1 (PD-L1), which effectively hampers the activity of T (or CAR–T) cells and NK cells expressing the corresponding PD-1 receptor through immune checkpoint activation [28]. The activation of MDSCs induces the generation of reactive oxygen species/nitrogen species (ROS/RNS) and triggers the production of peroxynitrite (PNT) metabolites via STATA3 phosphorylation. This process leads to impaired functional T cell antigen recognition, compromised cell recruitment and infiltration ability, as well as direct cytotoxicity towards T cells [27]. MDSCs also utilize the exonucleases CD39 and CD73 to convert adenosine triphosphate (ATP) into adenosine, resulting in the loss of activation, proliferation, and tumor chemotaxis of immune effector cells. This leads to a shift towards immunosuppressive cell populations that ultimately suppress tumor immunity [28]. Studies have demonstrated the presence of MDSC-derived exosomes possessing inhibitory activity within the body, which can shape other immunosuppressive populations in the TME and ultimately result in functional CAR–T cell failure [74]. Tumino et al. demonstrated *in vitro* that PMN-MDSCs significantly suppress the anti-tumor cytotoxicity of disialogganglioside CAR–T (GD2-CAR–T) cells [75]. Gene expression profiling of PMN-MDSC-treated GD2-CAR–T cells revealed downregulation of genes associated with cellular activation, signaling, inflammation, and cytokine/chemokine secretion. The expression levels of these genes were found to be significantly correlated with patient prognosis. Furthermore, in neuroblastoma (NB) patients treated with GD2-CAR–T cells, the frequency of PMN-MDSCs in the bloodstream exhibited an inverse correlation with the abundance of GD2-CAR–T cells. Notably, this inverse correlation was more pronounced in patients who showed no response or experienced a loss of response to treatment, thereby providing compelling evidence for the inhibitory impact exerted by MDSCs on CAR–T cell function.

Therefore, the aforementioned studies suggest that MDSCs utilize diverse and non-redundant pathways to suppress T or CAR–T cell-mediated immune responses in cancer, which represents a critical determinant of tolerance towards CAR–T therapy.

#### Relevance of regulatory T cells in modulating the functionality of CAR–T cells

Tregs represent a heterogeneous subset of CD4^+^ T cells, and their expression of the Foxp3 protein serves as a lineage-specific transcription factor, which is utilized as one of the markers for lineage differentiation due to its absence in effector T cells (Teff). FOXP3 is commonly regarded as the “master regulatory transcription factor” of Treg cells, but its expression in activated conventional CD4^+^ T cells and absence in the highly suppressive inducible Treg (iTreg) subset of TME are inconsistent with this definition [76]. Based on the co-expression levels of FOXP3 and CD45RA, a marker of naive T cells, two distinct subsets of Tregs have been defined by some researchers: CD4^+^CD45RA^+^FOXP3^low^ naive or nTreg with weak suppressive function, and CD4^+^CD45RA^−^foxp3^high^ effector cells or eTregs that exhibit strong suppressive function and represent true suppressive Tregs [77]. Furthermore, there exists a subset of CD4^+^CD45RA^-^FOXP3^low^ cells that do not possess regulatory T cell properties and instead produce proinflammatory cytokines such as IFN-γ [77]. Upon TCR stimulation, nTreg undergo proliferation and differentiation into highly suppressive eTreg [78].

It is widely acknowledged that the immune system comprises a diverse array of lymphocytes and myeloid cells, which synergistically maintain immune homeostasis by facilitating host’s augmentation of protective responses against foreign entities or self-mutated proteins, while concurrently mitigating detrimental reactions towards self-antigens. In contrast to conventional CD4^+^ T helper (Th) cells, Tregs possess the unique ability to suppress immune responses in order to mitigate potential deleterious effects caused by Th cells. These functions are integral to maintaining immune homeostasis and preventing excessive activation of the immune system, which is crucial in tumor diseases where locally infiltrating Tregs inhibit anti-tumor specific immune response through various pathways including co-inhibition of receptor signaling, promotion of tumor immune escape, and facilitation of tumor growth. An elevation in the levels of circulating or tumor-infiltrating Tregs has been linked to unfavorable patient outcomes in various types of cancer, such as breast cancer, melanoma, and lung cancer [79]. However, the efficacy of CAR–T cell therapy in targeting Treg-infiltrated solid tumors has fallen short of expectations [80]. The mechanism of Treg-mediated immunosuppression is primarily associated with the following factors: (1) TGF-β, as intratumoral Tregs exhibit elevated levels of secreted TGF-β within the tumor microenvironment [81]. Tregs regulate their migration and retention in inflamed tissues via the TGF-β pathway, including GPR15-mediated homing to the colon mucosa [82]. Mechanistically, the activation of TGF-β signaling facilitates the interaction between Smad3 and *CNS1*, which is the enhancer region of Foxp3, thereby governing the regulation of Foxp3 expression [83]. Furthermore, the cooperative function of Smad2 and Smad3 is crucial in the generation of induced regulatory T (iTreg) cells [84]. Therefore, TGF-β plays a crucial role in mediating FoxP3 induction, promoting Treg differentiation and maintenance, as well as enhancing the inhibitory activity of Tregs. *In vitro* studies demonstrated that activated Tregs effectively secreted active TGF-β1 through the transmembrane protein glycoprotein-A repetitions predominant (GARP) [85]. The active form of TGF-β1, which is membrane-bound, exerts an immunosuppressive effect on Teffs through direct cell contact [86]. It was demonstrated that *in vivo*, GARP mab could effectively suppress the immunosuppressive activity of Tregs [87]. The significance of TGF-β1 in Tregs and its immunosuppressive function is underscored by this finding, highlighting the potential of GARP as a promising therapeutic target for augmenting anti-tumor immune responses through Treg-induced activation of TGF-β1 and enhancement of Treg suppressive function in cancer. (2) ADO: Studies have demonstrated that human iTreg upregulate the surface expression of CD39 and CD73, efficiently hydrolyzing ATP into 5’-AMP and ADO, while actively secreting ADO, which accumulates in the cellular periphery. The autocrine pathway involving ADO appears to enhance Treg stability and function. In Teff cells, where A2AR is also expressed, the activation of adenosine signaling pathway leads to a CAMP-mediated downregulation of cellular function [88]. (3) Encompassing additional Treg-mediated immunosuppressive mechanisms within the TME, as outlined in Table 2.

**Table 2:** Mechanisms used by Treg for immune suppression in the TME

Mechanism	Reference
Inhibitory cytokine production:	
IL-10, TGF-β, IL-35	[89,90,91]
Soluble inhibitory factors:	
IDO, PGE_2_	[90,92]
T effector cell cytolysis:	
Granzyme B, perforin	[93,94]
Metabolic interruption:	
IL-2 deprivation	[95]
Adenosine production	[90,96]
cAMP-mediated effects	[97]
Receptor/ligand signaling:	
Fas/FasL, TGF-β/TGFRII	[90,98]
Checkpoint Inhibition:	
CTLA4, PD-1, TIM-3, LAG-3, TIGIT	[99]
Exosomes	[100,101]

Exploiting the crucial immunosuppressive role of Treg in the TME, therapeutic strategies targeting the elimination or reduction of Treg have demonstrated significant enhancements in anti-tumor efficacy. Previous studies have demonstrated that CD28-CD3ζ-CAR–T cells are more effective in inducing infiltration of Treg cells into tumors compared to CD3ζ-CAR–T cells. Knockout of the lck binding region within the CD28 domain, which is associated with IL-2 production, reversed the induction of Treg cell infiltration into tumors and enhanced the anti-tumor activity of CAR–T cells [80]. In patients with melanoma, administration of high doses of interleukin-2 leads to an elevation in the population of circulating CD4^+^CD25^+^Foxp3^+^ regulatory T cells [102]. The presence of CD4^+^Foxp3^+^ cells exerts a negative impact on adoptive immunotherapy and immune response. Transient depletion of regulatory T cells (Tregs) using IL-2 diphtheria toxin (IL-2DT) resulted in a reduction in tumor burden and enhanced proliferation of adoptively transferred CTLs specific to the AML tumor, as demonstrated in an *in vivo* model [103].

Collectively, these findings indicate that Tregs within the tumor microenvironment exert a significant inhibitory effect on anti-tumor immunity and represent a crucial factor contributing to the tolerance and long-term efficacy of CAR–T cell therapy. Therefore, strategies aimed at counteracting the immunosuppressive function of Tregs or reducing their abundance may enhance the therapeutic potential of adoptive CAR–T cells.

#### The impact of cancer-associated fibroblasts (CAF) on the functionality of CAR–T cells

Cancer-associated fibroblasts (CAFs) are a heterogeneous group of activated fibroblasts, constituting the main component of tumor stroma and playing a crucial role in the TME. The phenotypic alterations of CAFs significantly impact tumor progression and treatment response. CAFs exert their influence by regulating various biological functions within the tumor stroma, including immune modulation, angiogenesis, extracellular matrix remodeling, as well as generation and maintenance of cancer stem cells. Consequently, they contribute to the development of treatment resistance. However, due to the absence of universally applicable biomarkers for identification purposes, there is currently no standardized or consensus-based approach for characterizing CAFs. Presently, CAFs are defined as cells lacking expression of epithelial-, endothelial-, or hematopoietic-specific markers while expressing mesenchymal markers such as vimentin, α-smooth muscle actin (α-SMA), fibroblast activation protein (FAP), and platelet-derived growth factor receptor α (PDGFR-α), without any accompanying gene mutations [104]. CAFs play a pivotal role in shaping the tumor microenvironment by recruiting, promoting, or collaborating with various immunosuppressive cells. Additionally, CAFs secrete an array of immunosuppressive factors and establish a microenvironment that facilitates the survival and proliferation of tumor cells. The formation of an immunosuppressive tumor microenvironment has been demonstrated to be mediated by CAFs through the secretion of a diverse array of cytokines, growth factors, chemokines, exosomes, and other effector molecules. This intricate communication network enables cancer cells to evade immune surveillance and imposes limitations on the efficacy of immunotherapy strategies. For instance, the expression of ligands CXCL12 [105], CXCL1, and G-CSF by CAFs can induce downstream immunosuppressive signaling pathways. Among them, CXCL12 facilitates the recruitment of immunosuppressive cells and their precursors in the tumor microenvironment, particularly bone marrow-derived mesenchymal stem cells and monocytes that differentiate into TAMs. CAFs impede the activity and recruitment of CD8^+^ cytotoxic T cells within the tumor, partly mediated by TGF-β [106] and CXCL12. TGF-β and CXCL12 have been reported to enhance the rejection of cytotoxic T cells by attenuating the anti-PD-L1 response [105]. While suppressing antitumor cytotoxic T cells, CAFs can also enhance the recruitment of Tregs within the tumor microenvironment. Single-cell RNA sequencing analysis revealed an upregulation of PD-1 and CTLA4 in Tregs. CAFs appear to attract, aggregate, and support the survival of FOXP3^+^ Tregs in human triple-negative breast cancer [107]. Treg and CAFs, two distinct cell populations, are abundantly distributed within the interstitial region and have been correlated with unfavorable prognosis in various malignancies including lung cancer [108]. CAF-mediated recruitment of MDSCs to the tumor microenvironment via CCL2 impedes CD8^+^ T cell proliferation and IFN-γ production, thereby exerting immunosuppressive effects [109]. TAMs and CAFs exhibit synergistic effects and are frequently co-localized within tumor tissue regions. Their combined presence in human cancers serves as a negative prognostic indicator. FAP, a type II serine protease located on the surface of CAFs, is upregulated in numerous tumor microenvironments and serves as one of the molecular markers for CAFs. FAP plays a crucial role in the regulation of T cells and CAR–T cells across various tumor types. Anti-human flat-foot protein-positive FAP+CAF, derived from breast cancer patients, exhibits enrichment at the periphery of tumors where it closely interacts with T cells, thereby exerting an inhibitory effect on T cell proliferation through a nitric oxide-dependent mechanism [110]. Ersek et al. demonstrated that FAP^+^CAF impeded the NF-κB signaling pathway in CD8^+^ T cells, thereby suppressing the initial activation and cytotoxicity of CTLs [111]. The up-regulation of the forkhead box P3 (*FOXP3*) gene by FAP facilitates the infiltration, proliferation, differentiation, and immunosuppressive function of Treg cells [112]. Hou et al. observed a positive correlation between the expression level of FAP and the extent of infiltration of CD4^+^ CD25^+^ Treg cells in the stromal region of ovarian cancer tissues [113]. Additionally, the immunosuppressive effect of FAP on tumor immunity mediated by Treg cells is modulated by distinct subsets of CAFs. Costa et al. categorized CAFs into four subsets in triple-negative breast cancer, among which the FAP^+^ CAF subset (CAF-S1) is associated with Treg cell recruitment. Furthermore, B7-H3, CD73, and dipeptidyl peptidase-4 facilitate the differentiation of CD25^+^ T cells into FOXP3^+^ Treg cells while inhibiting effector T cell proliferation [114]. Kieffer et al. further classified breast cancer CAF-S1 into eight clusters and demonstrated that the FAP^+^CAF-S1 subpopulation enhances the expansion of CAF cells characterized by TGF-β pathway through activation of the nuclear factor of activated T cells (NFAT)/STAT pathway, leading to upregulation of PD-1 and CTLA-4 expression in Treg cells [107]. The aforementioned studies suggest that FAP functions as an immune escape mechanism by facilitating the interaction between CAF and Treg cells, thereby impacting adoptive cellular immunotherapy. Preclinical tumor models, including malignant pleural mesothelioma, melanoma, colon cancer, and breast cancer have demonstrated the efficacy of CAR–T cells targeting FAP [115].

#### The interactions between CAR–T cells and the tumor microenvironment

The TME exhibits immune escape and immunosuppressive characteristics, thereby contributing to the limited efficacy of CAR–T cells in solid tumors. As previously mentioned, within the TME, tumor cells along with Tregs, MDSCs, TAMs, CAFs, and their secreted inhibitory cytokines collectively orchestrate immunosuppressive effects. The microenvironment of solid tumors is highly intricate, leading to varying degrees of inhibition on the infiltration, activity, and function of CAR–T cells. This immunosuppressive microenvironment primarily consists of mesenchymal cells such as MDSCs, Tregs, CAFs, and TAMs; immune checkpoints like PD-L1; as well as a diverse range of tumor-promoting and immunosuppressive soluble factors including TGF-β. These factors are considered pivotal in influencing the activity and function of CAR–T cells. These factors can induce competition between CAR–T cells and tumor cells for oxygen and other nutrients in the hypoxic microenvironment, challenge the tolerance of acidic metabolic conditions, and impair the functionality of CAR–T cells. Currently, it is evident that CAR–T cells do not act autonomously in solid tumors; instead, they acquire their corresponding functional activity by directly interacting with various cells within the TME or relying on cytokine-mediated crosstalk. This phenomenon constitutes the primary factor contributing to the limited efficacy of CAR–T cell therapy in treating solid tumors.

Firstly, cellular interactions can induce immune evasion through the interaction of cell membrane surface proteins (ligands and receptors). For instance, the binding between PD-1 molecules on CAR–T cells’ surface and PD-L1 expressed on tumor or suppressor cells can lead to adoptive cell exhaustion and apoptosis, enhanced secretion of suppressor cytokines, promotion of immunosuppressive cell generation, and induction of T-cell transformation into Tregs. Additionally, exhausted CAR–T cells may exhibit overexpression of PD-1. Furthermore, upregulation in the expression of PD-L1 on tumor cells’ surface was observed [116]. Other checkpoint molecules, such as T-cell immunoglobulin and mucin domain-3 (TIM-3), cytotoxic T lymphocyte-associated antigen-4 (CTLA-4), and Lymphocyte Activation Gene 3 (LAG-3), also modulate the anti-tumor activity of CAR–T cells through their own immune regulatory pathways [117]. In this milieu, the efficacy of CAR–T cells in eradicating tumors is diminished, impeding their ability to effectively eliminate target antigenic cells. Prolonged exposure to antigens induces comprehensive transcriptomic and epigenetic reprogramming of CAR–T cells, leading to the development of exhausted CAR–T cells that detrimentally impact their anti-tumor functionality. Singh and colleagues conducted a comprehensive investigation into the exhausted CAR–T cell mechanism by employing a CRISPR-based unbiased genome-wide loss-of-function screen in the ALL cell line Nalm6. The study revealed a significant enrichment of targeted genes associated with the pro-apoptotic death receptor signaling pathway, including *FADD*, *BID*, *CASP8*, and *TNFRSF10B*, in CAR–T19 resistance. Conversely, guides targeting anti-apoptotic molecules such as *CFLAR, TRAF2*, and *BIRC2* were found to be depleted [118]. From a fundamental biological perspective, this implies that CAR–T cells, akin to T cells possessing natural T-cell receptors, may still exert cytotoxicity through intercellular membrane-protein interactions to activate either the intrinsic apoptotic pathway (via secretion of cytotoxic molecules such as granase and perforin) or the extrinsic apoptotic pathway (via activation of death receptors on the cell membrane). Furthermore, in the presence of chronic antigen exposure, these cells can acquire exhaustion-like characteristics.

Furthermore, within the TME, diverse cellular components engage in intercellular communication and elicit adaptive responses via autocrine or paracrine mechanisms mediated by cytokines, exosomes, or local metabolites, thereby influencing the viability and functionality of CAR–T cells. For instance, prostaglandin E2 (PGE2), a bioactive lipid frequently upregulated in tumors, exerts regulatory control over cell proliferation, migration, apoptosis, and angiogenesis to promote tumor survival. In CAR–T cell therapy, the activation of protein kinase A (PKA) through PGE2 and adenosine inhibits CAR–T cell signaling and activation, leading to a reduction in both cell proliferation and effector function [119]. Vascular dysfunction commonly observed in the TME often results in localized hypoxia and metabolic disturbances, thereby impeding the efficacy of CAR–T cell therapy [120]. In the hypoxic environment, CAR–T cells face competition with tumor cells for limited oxygen and nutrients, as well as the challenge of an acidic metabolic environment, resulting in impaired CAR–T cell function and ultimately treatment failure. Regarding autocrine and paracrine effects, the production of IFN-γ by CAR–T cells not only augments the activity of endogenous T cells and natural killer cells but also plays a crucial role in sustaining the cytotoxicity of CAR–T cells, as evidenced by *in vivo* imaging studies. IFN-γ secreted by CAR–T cells can promote the production of interleukin-12 and enhance the body’s immune response and CAR–T cell response [121]. The secretion of IFN-γ by CAR–T cells can also elicit a cascade of alterations in the TME, including modulation of neoangiogenesis [122]. However, armored CAR–T cells expressing IL-12, IL-15, IL-18, and IL-36γ were found to improve the persistence of CAR–T cells, reduce the expression of PD-1 markers of the exhaustion effect, recruit endogenous T cells and induce epitope spreading, promote host immunity, and improve tumor clearance *in vivo* [123]. T cells that constitutively coexpress CD19-targeting CARs along with IL-2, IL-7, IL-15, or IL-21 have also demonstrated enhanced *in vivo* tumor control [124]. However, constitutive overexpression of immune-stimulating cytokines also augments host toxicity. The cytokine release syndrome (CRS) is widely acknowledged as a severe adverse event associated with CAR–T cell therapy, wherein CAR-related CRS is characterized by the secretion of IL-1 and IL-6 by myeloid cells, with particular emphasis on the pivotal role of IL-6 in its pathogenesis [125]. The design of CAR–T cells constitutively expressing the membrane-bound IL-6 receptor (mbaIL6) effectively mitigates IL-6 signaling and function, ameliorating adverse reactions associated with CAR–T cell therapy while simultaneously exerting potent anti-tumor activity [126].

In conclusion, adoptive CAR–T cell therapy profoundly remodels the TME through direct or indirect mechanisms during host interaction, thereby influencing the efficacy of CAR–T cell therapy. Notably, by augmenting bystander cytotoxicity, this interaction significantly disrupts the carefully orchestrated proliferation niche of tumor cells, potentially synergizing with other anti-solid tumor therapies to overcome the immunosuppressive TME.

### The heterogeneity of tumor-associated antigens significantly impacts the efficacy of CAR–T cells

Despite the unprecedented clinical success observed in certain hematologic cancer types, CAR–T cells exhibit limited long-term efficacy in clinical treatment [4]. One of the primary factors contributing to this phenomenon is that CARs specifically target a single TAA, which typically recognizes only one specific molecule expressed on tumor cells. However, this approach proves ineffective in cases where tumors exhibit heterogeneous TAA expression or antigen-loss variants, ultimately leading to the development of drug resistance. Therefore, the therapeutic potential of CAR–T cell therapy may be constrained by tumor immune evasion resulting from antigenic loss. For instance, complete remission was achieved in nearly 90% of patients with relapsed or refractory B-cell acute lymphoblastic leukemia (R/R B-ALL) within one month following the administration of CAR–T19 cells; however, a considerable proportion of patients experienced subsequent relapse [127]. One of the primary mechanisms involves the apparent reduction in CD19 protein levels resulting from gene splicing, frameshift mutations, or deletions. Studies have revealed that one of the two copies of the CD19 gene on chromosome 16 undergoes deletion due to various factors. Typically, the remaining copy experiences a deletion or mutation in exon 2’s coding region, which is responsible for recognizing the CD19 epitope, leading to impaired sequestration of CD19 protein within the endoplasmic reticulum. The mutation or deletion of exon 2 leads to the generation of modified CD19, which exhibits enhanced stability compared to standard CD19 but fails to be recognized by CAR–T19 cells. An additional mechanism involves frequent exon skipping of exons 2, 5, and 6 in the patient’s gene splicing process, leading to premature termination of CD19 protein expression due to the absence of exons 5 and 6 [128]. Furthermore, lineage switching represents a plausible mechanism underlying resistance to CAR–T19 therapy [129]. For instance, Gardner et al. reported that among seven patients with mixed lineage leukemia (MLL) B-ALL who received CAR–T19 cell therapy, two experienced CD19-negative relapses due to the conversion of ALL to the AML lineage shortly after treatment [129]. In contrast to B-ALL and other hematological malignancies, solid tumors exhibit distinct variations in the intensity and distribution of antigen-positive cells, as well as antigenic heterogeneity within different tumor types or even within the same tumor, thereby presenting a formidable challenge for effective implementation of CAR–T cell therapy. Walker et al. discovered that the density of anaplastic lymphoma kinase (ALK) on neuroblastoma cell lines was suboptimal for achieving maximal activation of CAR–T cells, leading to limited efficacy of ALK CAR–T in two human neuroblastoma xenograft models [130]. Additionally, ALK CAR–T cells demonstrate prompt and complete internalization of T cell surface receptors upon antigen stimulation. By utilizing a model that regulates both antigen density and CAR expression, it has been demonstrated that the function of CAR is influenced by both target antigen and CAR density. Insufficient expression of ALK CAR leads to limited antitumor efficacy. Other studies have reported variable levels of Mesothelin (MSLN) expression in different tumor cells of the same patient, despite its high expression in non-small cell lung cancer (NSCLC) compared to normal tissues [131]. Compared to NSCLC, pleural mesothelioma and pancreatic cancer (PDA) cells exhibited a relatively higher percentage and intensity of MSLN expression among various tumor types [131]. Due to low expression on normal tissue and high expression on tumor tissue, HER2 is a frequently targeted TAA in solid malignancies. In an investigation involving patients with advanced NSCLC, immunohistochemical analysis revealed varying staining intensities of HER2 overexpression in 40% of tumor samples [132]. Furthermore, the presence of tumor heterogeneity has also been documented in other therapeutic targets for CAR–T cell therapy, including MUC1, PSCA, and epithelial cell adhesion molecule (EpCAM) [133]. Moreover, the presence of multiple combinations of TAA within a single tumor simultaneously adds complexity and heterogeneity to the composition and structure of TAA, thereby posing significant challenges for standardizing CAR–T therapy [134].

### Challenges pertaining to the quality control of cell isolation and culture conditions during CAR–T preparation process

The CAR–T cell generation protocol significantly influences the efficacy of CAR–T cells. In the context of chimeric antigen receptor therapy, patient-derived T cells are isolated and activated, genetically modified, and expanded for 9-11 days under nutrient-rich conditions prior to their utilization in clinical reinfusion therapy. In this process, dysregulation of T cell function in leukapheresis products may occur due to various factors such as prior treatment influence, variations in cell screening and separation standards, overstimulation during manufacturing, or differences in culture conditions. These factors can significantly impact the functionality and clinical efficacy of CAR–T cells. For instance, Fraietta and colleagues discovered that the functional phenotype of T cells in the leukapheresis product had an impact on the quality of resulting anti-CD19 CAR–T cell therapy in patients with chronic lymphocytic leukemia (CLL), thereby establishing a correlation with clinical efficacy [135]. Due to the heterogeneous characteristics of tumors, it is challenging to establish a standardized immunotherapy strategy across different TMEs, and the generalization of isolation, selection, and culture conditions for CAR–T cell subsets in personalized cellular products remains elusive. Revealing the process standards for CAR–T preparation poses a formidable challenge. The establishment of cell manufacturing protocols that are safe, efficient, robust, and cost-effective is crucial for subsequent clinical applications.

#### Origin, activation, and expansion of adoptive cells

The optimal composition of CAR–T cell products for the treatment of different hematologic or solid tumor types remains uncertain. During the process of CAR–T cell preparation, various operational methods and steps such as cell sourcing, activation, and expansion exert a significant influence on the final product’s functionality and clinical efficacy. Therefore, it is crucial to consider an implementation plan and steps that can effectively balance and enhance the CAR–T cell preparation process. The specific factors influencing this process are described as follows.

##### Impact of cell source on the functional efficacy of CAR–T preparation

The efficacy of CAR–T cells heavily relies on the quality and characteristics of T cells. The selection of an appropriate source constitutes the initial and pivotal step towards achieving successful CAR–T cell therapy. The CAR–T cells can be categorized as autologous or allogeneic (autoCAR–T or alloCAR–T) based on the origin of the T cell population. Both approaches have their merits and drawbacks. autoCAR–T cells exhibit superior therapeutic efficacy compared to alloCAR–T cells, with relatively fewer adverse effects and prolonged *in vivo* persistence. However, the utilization of autologous T cells is a time-consuming process that heavily relies on the quality and quantity of T cells obtained from patients, making preparation costly and lengthy. Consequently, it fails to meet the urgent treatment requirements for clinically acute and critical patients. The establishment of an appropriate autologous T cell generation protocol is widely recognized as the primary obstacle hindering its extensive clinical implementation. The major challenges faced by alloCAR–T cells are host-*vs*-graft disease (HvGD) and graft-*vs*.-host disease (GvHD). In the realm of future cancer immunotherapy research, it is worth exploring the potential of CRISPR genome editing tools to genetically manipulate TCR and human leukocyte antigen (HLA) in alloCAR–T cells, thereby mitigating these adverse effects. Moreover, T cell-derived induced pluripotent stem cells (iPSCs) have been demonstrated as an optimal source of autologous CAR–T cells that do not elicit GvHD, thereby facilitating the large-scale development of effective personalized CAR–T cell immunotherapies [136]. AutoCAR–T or alloCAR–T can be derived from two distinct cellular pathways: (1) tumor infiltrating lymphocytes (TILs); and (2) the presence of conventional α/βT lymphocytes in peripheral blood circulation. Although early studies predominantly focused on TILs in the published clinical data, their isolation from clinical tumor tissues poses challenges due to limited availability and slow expansion rate, thereby impeding clinical treatment. Recently, multi-center clinical trial reports have demonstrated that tumor infusion of autologous gene peripheral blood-derived T cells modified with CD19-specific CAR, following *in vivo* lymphofine preclearance treatment of the host, also elicits a robust anti-tumor response, consistent with patients treated with expanded TILs *in vitro* [137]. Furthermore, the application of GD2-specific CAR–T therapy derived from peripheral blood has demonstrated remarkable anti-tumor efficacy in neuroblastoma patients with solid tumors, obviating the need for pretreatment [138]. These clinical findings suggest that the therapeutic efficacy of CAR–T cells derived from TIL or peripheral blood circulation is not predominant, potentially due to their susceptibility to diverse immunomodulatory factors within the TME. Subsequent investigations have demonstrated the significance of exploring diverse subsets, including CD4^+^/CD8^+^ and naive/central memory/effector memory/end-effector populations, to enhance the clinical efficacy of CAR–T therapy [139]. The cytotoxicity of both CD4^+^ and CD8^+^ T cell subsets against tumor cells is significant. In contrast to the utilization of T cell subsets alone as CAR–T cells, the combination of CD4^+^ and CD8^+^ subsets exhibited synergistic anti-tumor effects both *in vitro* and *in vivo*. Maintaining a balanced composition of CD8^+^ and CD4^+^ CAR–T cells is advantageous for effective targeting of solid tumors. In the context of CAR–T cells, CD4^+^ T cells exhibit comparable direct antitumor activity to cytotoxic CD8^+^ CAR–T cells [140,141]. In the context of glioblastoma, CD4^+^ CAR–T cells exhibited superior anti-tumor activity compared to CD8+ CAR–T cells, particularly in terms of long-term anti-tumor response [141]. The synergistic anti-tumor effect of CD4^+^ helper T cells and CD8^+^ cytotoxic T cells at a 1:1 ratio significantly enhances tumor eradication efficiency. Furthermore, CD19 CAR–T cell therapy has demonstrated a remarkable remission rate among patients with B-ALL. The antitumor activity and long-term persistence of poorly differentiated CD19 CAR–T cell products, which are enriched in Naive and central memory T cells, have been demonstrated to be superior in both preclinical and clinical studies conducted on hematologic malignancies [142]. The CD8^+^ and CD4^+^ CAR–T cells derived from naive and central memory T cells exhibited superior performance compared to effector memory T cell-derived CAR–T cells. However, the findings in solid tumors did not demonstrate consistent outcomes compared to those observed in hematologic malignancies. Csaplar et al. conducted a comprehensive *in vitro* and *in vivo* comparison to evaluate the effector functions of poorly differentiated (T_cm_-enriched) and highly differentiated (T_em_-enriched) HER2-CD28z and HER2-41BBz CAR–T cell products [143]. The enhanced effector functions of T_em_-enriched HER2-CAR–T cells were demonstrated *in vitro*, including their superior clonal expansion observed in repeated stimulation assays. The *in vivo* antitumor activity and expansion capacity were concurrently enhanced. Subsequent investigations revealed that, in the presence of a fixed target antigen, the enriched products containing effector memory T cells exhibited augmented secretion of IFNγ and IL-2, thereby inducing more potent CAR-specific anti-tumor activity. The cytolytic activity *in vitro*, anti-tumor activity *in vivo*, and expansion ability of CD28-z-CAR–T cells surpassed those of 41BB-z-CAR–T cells [143]. The higher activation of highly differentiated T cells by the CD28 costimulatory construct is attributed to its superior efficacy compared to 41BB [144]. In contrast to studies targeting CD19 leukemia using CAR–T cells, the disparate outcomes observed in this study can be attributed to several factors, such as variations in the targeted antigen [145] or differences in the molecular composition of solid tumor cells compared to leukemia cells. Furthermore, leukemic blasts exhibit enhanced susceptibility to circulating CAR–T cells due to their accessibility and lack of defense mechanisms, in contrast to the intricate tumor microenvironment observed in solid tumors. The latter comprise a densely packed extracellular matrix that serves as both a physical barrier [146] and houses CAFs, tumor-infiltrating macrophages, as well as regulatory T and B cells, all of which possess the potential to negatively regulate immune responses [78]. These factors have distinct threshold requirements for CAR–T to elicit anti-tumor activity, and it is evident that CAR–T composed of highly differentiated effector T cells facilitates the attainment of these conditions and exerts a superior anti-tumor effect. The sensitivity of different tumor types to the composition of CAR–T subsets based on tumor heterogeneity may vary, suggesting a need for analysis or detection of tumor-sensitive T cell subsets and rational screening of differentiated subsets during the preparation process to achieve optimal combination and enhance clinical efficacy.

##### Repercussions of cellular activation and expansion on the functionality of engineered CAR–T cells

The primary metabolic pathways and energy acquisition mechanisms in naive T cells (TN) involve OXPHOS and mitochondrial fatty acid oxidation (FAO). Upon receiving stimulatory signals from CD3/CD28 antigens, T cells enhance their metabolic rate to meet the heightened biosynthetic demand. It modulates metabolic signaling to enhance aerobic glycolysis, known as the Warburg effect. This metabolic reprogramming also induces the conversion of TN cells into Teff cells [147]. Given the potential impact of culture conditions and procedures on T cell differentiation, which subsequently influences the persistence and clinical efficacy of CAR–T cells, a comprehensive investigation is warranted. In the context of T cell activation and expansion, IL-2 has traditionally served as the benchmark cytokine for CAR–T cell culture. IL-2 can rapidly induce T cell proliferation *in vitro*, promote the shift from oxidative phosphorylation to glycolysis, enhance effector T cell formation, and decrease memory T cell population. However, IL-2 can induce Fas-mediated apoptosis of T cells, leading to an increased propensity for early apoptosis in IL-2-stimulated cells, thereby exerting a detrimental impact on long-term T cell toxicity. The conjugation of RetroNectin (a recombinant human fibronectin fragment and T cell proliferation stimulating factor) with OKT3 (an anti-CD3 monoclonal antibody for activated T cells) significantly augmented the expansion of T cells compared to standard OKT3-antiCD28 activation, while effectively preserving the phenotypes of T cell naïve and central memory [148,149]. Gargett et al. observed that treatment of RPMI medium supplemented with IL-2 with OKT3/RetroNectin resulted in a higher abundance of CD45RA^+^ stem/memory subsets and a significant augmentation in CD8^+^ T cells [149]. The molecular mechanism may be associated with the phosphorylation of GSK3 [150]. The study conducted by Stock et al. demonstrated that RetroNectin-based activation in conjunction with a CD19-targeting third-generation CAR resulted in the enrichment of CD8^+^ cytotoxic and less differentiated naivelike (CD45RA^+^CCR7^+^) CAR–T cells [149]. Additionally, IL-7 and IL-15, which are other gamma-chain cytokines, were observed to enhance OXPHOS and suppress glycolysis in order to induce metabolic adaptation [151], resulting in a less differentiated T cell phenotype compared to IL-2. This metabolic modulation led to more durable and superior antitumor effects. Consequently, by employing various combinations of IL-2, IL-7, IL-15, OKT3 along with RetroNectin or AntiCD28 for cellular activation and expansion in complete RPMI medium, consistent expansions of distinct cell subsets at different stages of differentiation were achieved. Furthermore, the modulation of metabolites can also impact the differentiation extent of T cells. This phenomenon is primarily attributed to the regulation of T cell metabolism through modulating metabolites to enhance OXPHOS and appropriately suppress glycolysis. For instance, Sukumar et al. employed the competitive glucose inhibitor 2-deoxy-d-glucose (2-DG) to modulate the differentiation trajectory of CD8^+^ T cells during *in vitro* expansion, aiming to enhance the generation of memory cells and thereby promoting long-lasting anti-tumor functionality [152]. Furthermore, supplementation of L-arginine and carnosine, along with a reduction in glutamine levels, demonstrated enhanced *in vitro* lysis and improved elimination of tumor cells *in vivo* [153]. Therefore, in the case of heterogeneous tumors, this type of artificial activation amplification factor formulation or metabolic stimulation scheme appears to be more favorable for precisely inducing sensitive and targeted CAR–T cells, thereby providing robust support for enhancing the clinical treatment efficacy.

#### To optimize the manufacturing process of CAR–T cells for enhanced clinical efficacy and minimized adverse reactions

The implementation of Current Good Manufacturing Practice (cGMP) is crucial in ensuring the quality and compliance of cell product manufacturing processes. The production of clinical-grade cell products involves intricate processes that are rigorously regulated under cGMP and necessitate adequate cell manufacturing facilities, ancillary products, and manufacturing procedures to comply with the guidance requirements set forth by the Food and Drug Administration (FDA). Given the unique nature of autologous cell therapy products, manufacturers must integrate scientific knowledge pertaining to product definition with relevant drug regulations, tailoring each individual cell therapy product accordingly. This entails considering various aspects such as CAR–T cell design, manufacturing processes and steps, quality standards, detection methods, etc., in order to ensure optimal product quality while enhancing production efficiency and minimizing adverse reactions. The translation of CAR–T from the laboratory to the clinic requires a meticulously designed process for scaling up and validation. The validation of a process necessitates the establishment of scientific evidence to demonstrate its consistent capability in delivering a product of superior quality. The process verification can be categorized into three stages: the stage of process design, the stage of process confirmation, and the stage of continuous process confirmation. Ultimately, it is essential to conduct a release test and issue certificates for qualified analysis. Therefore, to ensure the clinical advancement of CAR–T therapy, it is imperative to integrate large-scale cGMP infrastructure with personalized CAR innovations in order to optimize production costs. Employing a cGMP-certified automated manufacturing platform would be an optimal choice.

### The influence of on target-off tumor adverse reactions on the clinical compliance of CAR–T cell therapy

The clinical research or application of CAR–T is often hindered by severe and potentially life-threatening toxicities, including CRS, graft-*vs.*-host disease (GVHD), on-targeted/non-tumor toxicity, neurotoxicity (immune effector cell-associated neurotoxicity syndrome, or ICANS), and tumor lysis syndrome, which significantly restrict its clinical utility. The most concerning adverse effects associated with CAR–T cell therapy include CRS and uncontrolled immune responses targeting healthy tissues with low TAA expression. The rapid activation and expansion of CAR–T cells result in CRS, characterized by significantly increased levels of soluble IL2, IL6, IL10, IFNγ, as well as elevated CRP and decreased ferritin and fibrinogen [5]. The incidence of CRS in patients receiving anti-CD19 CAR–T cell therapy has been reported to range from 54% to 91%, while severe CRS rates vary between 8.3% and 43%, depending on the specific CAR–T cell therapy product and grading system employed. CRS typically manifests within 1-6 days following CAR–T cell infusion and is characterized by pyrexia, often accompanied by other nonspecific influenza-like symptoms, hypotension, and/or hypoxia. If left untreated, CRS can rapidly progress to organ dysfunction; therefore, prompt administration of anti-cytokine therapy is crucial. Patients with more severe CRS typically exhibit an earlier onset and prolonged duration of CRS following CAR–T cell infusion [154]. In severe cases, additional adverse events may manifest, including capillary leak syndrome, vasodilatory shock, coagulopathy, and multiple organ failure. These events are accompanied by a cytokine profile akin to that observed in macrophage activation syndrome/hemophagocytic lymphohistiocytosis. Hong et al. observed a significantly augmented occurrence of CRS and elevated cytokine levels in patients with higher baseline tumor burden as determined by FDG PET/CT testing [155]. Giavirdis et al. demonstrated that CAR–T cells recruit and activate macrophages, resulting in the production of IL-6 and iNOS by myeloid cells, thereby contributing to CRS. Norelli et al. demonstrated that monocytes and macrophages were the primary contributors to the elevated levels of IL-1 and IL-6 in their mouse model of CRS, with IL-1 production preceding and potentially stimulating IL-6 production [125]. In a clinical trial evaluating CD19 CAR–T cell therapy for B-ALL, CLL, and NHL, researchers observed evidence of heightened endothelial activation in patients experiencing severe CRS, as indicated by elevated levels of von Willebrand factor and an increased Ang2:Ang1 ratio [154]. The activation of T cells expressed in normal tissues in response to TAA poses a significant safety concern for CAR–T cell products, potentially leading to targeted but non-neoplastic effects. Due to the non-exclusive expression of the targeted antigen on tumors, unexpected expression of target antigens in critical tissues can result in significant complications. The lack of tumor antigen specificity in CAR–T cells poses a clinical challenge for conventional cancer treatment, as it increases the risk of targeted non-tumor toxicity in normal tissues. For instance, the depletion of normal B cells following CD19 CAR–T cell therapy [156] or the occurrence of severe cholestatic hepatotoxicity observed in renal cancer patients during carbonic anhydrase IX therapy may be attributed to the recognition of target antigens expressed by normal biliary epithelial cells. Previous clinical trials have documented the cytotoxic effects of MART-1 and gp100-specific T cells on normal melanocytes, resulting in a majority of patients presenting with extensive erythematous rash, transient uveitis, or hearing loss; however, these adverse events were mostly ameliorated by topical steroid therapy [157]. However, in a separate trial, transgenic TCR-T cells targeting colorectal TAA CEA induced transient but severe colitis [158].

In summary, the occurrence of adverse reactions during tumor CAR–T therapy significantly impedes the clinical efficacy and long-term prognosis of CAR–T immunotherapy, posing a major obstacle to cellular immunotherapy. In recent years, researchers have developed various innovative designs aimed at reducing or regulating these adverse reactions and have made progress in numerous preclinical or clinical trials, opening up new possibilities for the clinical application of CAR–T immunotherapy.

**Figure 1:**
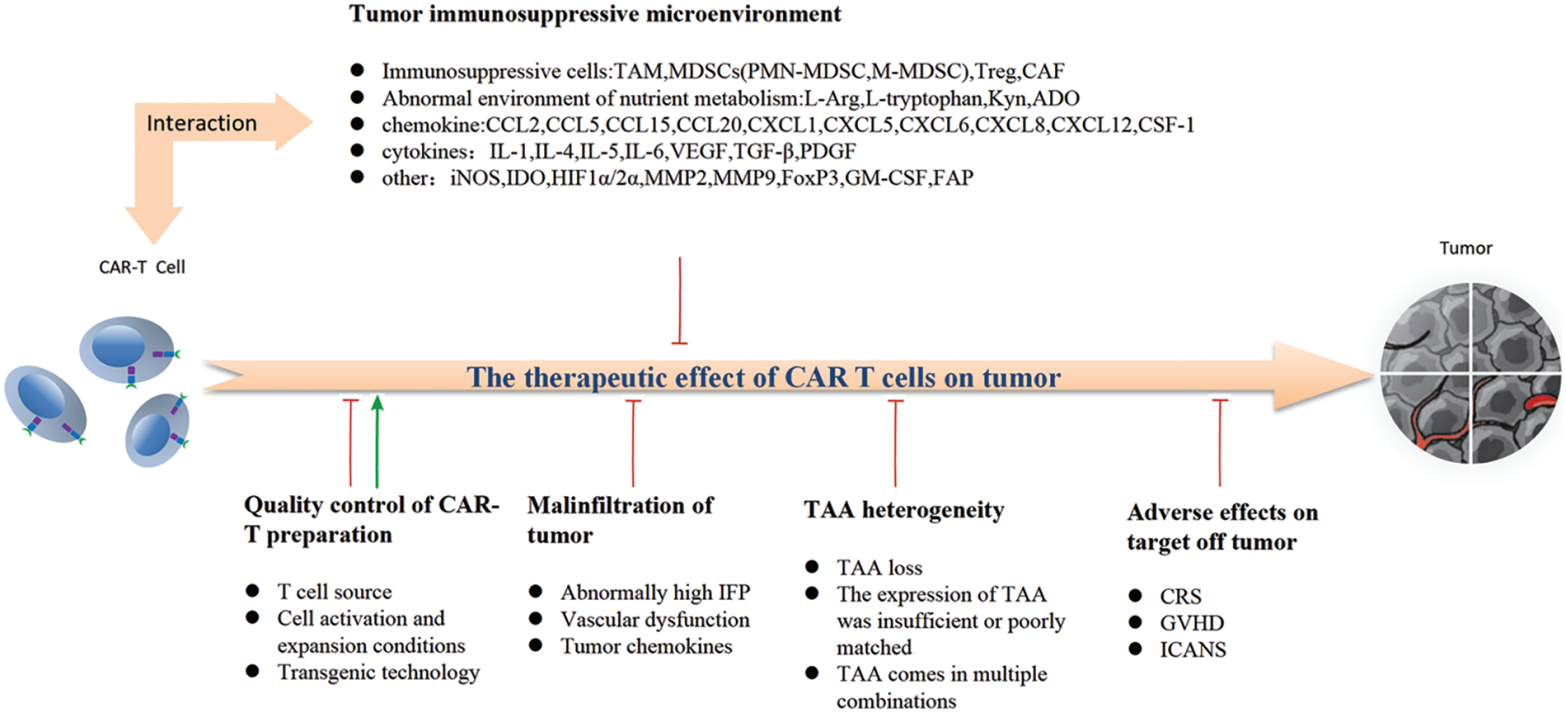
The efficacy of CAR-T targeting on tumor is mainly affected by the following aspects: The quality control of CAR-T preparation is a double-edged sword that affects the efficacy, and high-quality CAR-T can significantly improve tumor prognosis; The degree of tumor infiltration by CAR-T determines whether tumor cells can be directly targeted and eliminated. Heterogeneity of tumor-associated antigens leads to loss or mismatch of targeted antigens, resulting in drug resistance; The inhibitory effect of various immunosuppressive factors on the expansion and function of CAR-T cells in TME, as well as various adverse reactions of targeted tumor removal during CAR-T targeted therapy, resulted in the decline of patient compliance and affected the therapeutic effect.

The main influencing factors related to tumor efficacy of CAR–T therapy are shown in Fig. 1.

## Reinforcing the Antitumor Efficacy of CAR–T Cells: Strategies for Enhancement

Enhancing the clinical therapeutic efficacy of CAR–T cells has been a paramount objective pursued by diligent researchers. In recent years, a plethora of innovative optimization strategies have been employed in CAR–T cell research, yielding remarkable outcomes that hold significant practical implications for subsequent clinical investigations and applications. The ensuing sections provide a systematic classification and comprehensive exposition of these advancements.

### Optimizing the source and composition of CAR–T cells

The previous discussion has highlighted the significant impact of CAR–T cell source and subset composition on clinical efficacy. Regarding cell source, autoCAR–T cells exhibit immune rejection resistance. However, prolonged infiltration into the TME diminishes cytotoxicity and induces T cell exhaustion, potentially resulting in inadequate therapeutic effects of autoCAR–T cells against tumors. AlloCAR–T can be pre-collected and prepared, enabling timely administration to patients without the need for waiting during the preparation process. Additionally, T cells derived from healthy donors generally exhibit enhanced cytotoxicity, thereby potentially reducing costs and facilitating implementation. However, the major challenges in AlloCAR–T application lie in managing HvGD and GvHD. Theoretically, the utilization of gene editing technologies such as TALEN and CRISPR tool system to disrupt the normal expression of TCR and HLA genes holds potential for mitigating the aforementioned adverse reactions. However, clinical studies have revealed that AlloCAR–T exhibits a significantly shorter *in vivo* survival time compared to AutoCAR–T. Additionally, approximately 50% of patients who achieve complete remission after AutoCAR–T therapy still exhibit detectable CAR transgenes even after 1 year. In contrast, only one patient treated with AlloCAR–T displayed detectable CAR transgenes beyond 120 days, which could potentially contribute to the lower efficacy observed for AlloCAR–T [159]. In terms of adverse reactions, AlloCAR–T exhibited a higher incidence of CRS, while other adverse events demonstrated comparable frequencies between the two cohorts. Prolonged *in vitro* expansion of either AutoCAR–T or AlloCAR–T leads to T-cell exhaustion, resulting in diminished effector function. Additionally, the manufacturing process exhibits batch-to-batch variation, posing challenges for standardization and validation of the product. The urgent clinical application necessitates the development of third-party cell therapy products, which are artificially induced or derived from healthy donors, possessing distinct characteristics that are easily obtainable and widely applicable. To tackle the challenge of CAR–T cell production, human induced pluripotent stem cells (iPSCs) offer a replenishable cellular reservoir that can be genetically manipulated to differentiate into immune cells exhibiting augmented antitumor cytotoxicity. T cell-derived induced pluripotent stem cells (T_iPS_) hold great promise as a potential source for generating ‘off-the-shelf universal’ CAR–T cells; however, *in vitro* differentiation of T_iPS_ often yields T cells with suboptimal characteristics [136]. van der Stegen et al. discovered that premature expression of T-cell receptors or constitutive expression of CARs in T_iPS_ cells promotes the acquisition of innate phenotypes, which can be circumvented by disabling the TCR and relying solely on CAR signaling to drive differentiation. By delaying CAR–T expression and fine-tuning its signal intensity in T_iPS_ cells, human TCR^-^CD8αβ^+^ CAR–T cells similar to CD8αβ^+^ CAR–T cells found in peripheral blood were generated, effectively controlling tumor growth in a murine leukemia model without inducing GVHD. The utilization of CARs to drive T-cell maturation in T_iPS_, even in the absence of TCR, may facilitate the large-scale development of potent allogeneic CD8αβ^+^ T cells for a diverse range of immunotherapeutic applications [136]. Furthermore, unconventional T cells, including virus-specific T cells (VST), lipid-restricted (CD1) T cells, MR1-restricted T cells, and γδTCR T cells, possess inherent natural characteristics that make them a viable source for CAR cell production without the necessity of specific TCR or HLA gene editing [160]. The VSTs and unconventional T cells exhibit a memory-like phenotype, enabling rapid response to antigenic stimulation, thereby inducing potent cytolytic activity and cytokine production [161]. Several of these T cell subtypes have been effectively redirected towards tumors through genetic engineering of CARs or TCRs, while largely preserving their intrinsic characteristics. Retention of these cell-specific features may facilitate their *in vivo* re-expansion following adoptive cell transfer by exposure to their native cognate antigens, thereby enhancing the efficacy of immunotherapy [162]. Another advantage lies in the relatively restricted pattern of recognition exhibited by the endogenous TCR towards the target antigen. For instance, CD1-restricted T cells and γδ-TCR T cells possess the ability to identify ligands originating from altered tumor metabolism, thereby circumventing any potential harm to healthy cells [163].

In terms of the composition of CAR–T-derived cell subsets, the final clinical efficacy and prognosis are significantly influenced by the composition of CAR–T subsets targeting hematological tumors and solid tumors, considering the differences in clinical tumor heterogeneity and observed efficacy. This aspect has been previously discussed in detail and will not be reiterated.

### Enhanced design of CAR–T cell construction

#### Refinement and advancement of the classical CAR architecture

The principle of CAR–T cell tumor therapy is based on the mechanism of targeted recognition of tumor cells by genetically engineered T cell antigen receptors and subsequent activation of T cell-mediated immune clearance. The rationality of CAR design plays a crucial role in determining the functional activity of CAR–T cells, as they express antigen-specific non-MHC restricted receptors (CAR structure) through plasmid or viral vector gene recombination. Presently, the CAR structure comprises an extracellular antigen-recognition domain primarily derived from scFv of monoclonal antibodies. The term scFv refers to the heavy chain variable fragment of a specific antigen antibody, which is connected to the light chain variable fragment through a flexible linker (the heavy and light chains of the antibody are linked by a flexible linker). The single-stranded variable fragment is genetically linked to a hinge region, a transmembrane domain, an intracellular signaling domain, and/or a costimulatory molecule [3]. Some scFv were derived from established antibody clones, while others were selected from synthetic libraries. Among the selection screening methods, phage display is widely recognized as the most prevalent technique [164]. Additionally, there exists the CARbody approach, wherein scFv libraries are directly cloned into the CAR framework and subsequently expressed in primary T cells. These libraries are then selected based on their ability to activate cell-surface expressed targets on T cells [165]. In the process of constructing and screening CARs, it is imperative to consider a multitude of interconnections that impact CAR functionality. During the fusion of variable heavy chain (VH) and variable light chain (VL) fragments, optimization of biophysical properties becomes crucial in order to minimize aggregation, enhance conformational stability, and simultaneously preserve affinity towards the targeted antigen. The literature has extensively reviewed the factors that influence the accurate folding and aggregation of individual variable fragments and whole scFv, as well as the complementarity between different gene frames determining region loop grafting and stable mutation screening for domain optimization [166]. In addition to the amino acid composition and configuration of the variable fragment (VH-VL or VL-VH), the linker also exerts an influence on the thermodynamic stability of the single strand. Furthermore, it is imperative to consider the provenance of single-stranded Fv. Despite their widespread use, murine scFvs are prone to immune-mediated rejection, resulting in various consequences such as diminished CAR–T cell persistence and allergic responses [167]. In response to the limitations observed in the application of extracellular scFv, researchers have recently explored alternative targeting domains, including small-sized Fc-less molecules such as Camelid single-domain antibodies (VHH or nanobodies), peptides, and ligands (e.g., artificial biological orthogonal targeting). The VHHs, the smallest single-domain antibodies weighing 12-15 kDa, are half the size of scFv (25 kDa) and solely composed of VH variable domains. While nanobodies primarily interact with antigens through their complementary determining region 3 (CDR3), similar to full IgGs, their CDR3 region can adopt a stretched convex aileron conformation when binding to a concave epitope [168]. This property enables nanobodies to penetrate into crevices or inaccessible and enigmatic epitopes that are beyond the reach of conventional monoclonal antibodies. The incorporation of VHHS into CAR–T cells confers potent targeting ability, owing to their stable physical and chemical properties that enable them to withstand extreme conditions such as high pressure or acidity while maintaining a high affinity for antigens [169]. Furthermore, the absence of Fc and smaller molecular size, coupled with a high degree of sequence homology to the human *VH3* gene family, contribute to the relatively diminished immunogenicity of VHHs in humans [170], However, complete elimination of the induced anti-nanobody autoantibodies was not achieved [171]. CARs constructed using VHHs extracellular structures can overcome limitations associated with traditional single-chain antibody CARs, such as intricate folding and assembly processes, as well as reduced protein stability that may compromise functionality [172]. Hence, VHHs can serve as an optimal antigen recognition domain for the generation of CAR–T cells specific to tumor antigens [172]. Considering the distinctive structural and molecular characteristics of VHHs that facilitate their efficient infiltration into tumor tissues, harnessing VHHs for constructing T cell vehicles could potentially serve as a promising strategy. In terms of anti-tumor efficacy, Nasiri et al have demonstrated that VHH-CAR–Ts can effectively induce CD19-dependent tumor killing response, exhibiting comparable potency to its SCFV-based counterpart [173].

The humanization of TAA targeting domain holds distinct significance across various clinical applications. The authors, Jiang et al., successfully engineered a humanized hu8E5-2I scFv specific to CLDN18.2 antigen [174]. The Cldn18.2-specific chimeric antigen receptor (CAR) T cells were engineered utilizing the hu8E5-2I scFv as a targeted moiety. Remarkably, the hu8E5-2I scFv-CAR–T exhibited potent antitumor activity in both xenograft mouse models and patient-derived xenograft (PDX) models of ClDN18.2-positive gastric cancer. The humanized SCFV-structured CAR–T exhibits persistent *in vivo* presence and demonstrates effective tumor tissue penetration. The evidence demonstrates that human modification of scFv enhances its *in vivo* persistence and mitigates the occurrence of anaphylactic reactions. The authors, Temple et al. developed humanized CAR–T cells (H24 nanoCARs) based on the nanobody framework for patients who experienced recurrence following anti-CD19 CAR–T cell therapy. They targeted CD72, which is expressed in B-cell malignancies. H24 nanoCARs were compared to a nonhumanized parent (“NbD4”) CD72-nanometer CAR and clinically approved CD19-directed CAR–T constructed tissue cells [175]. The results revealed that H24 exhibits a significantly higher binding affinity towards CD72 and demonstrates enhanced efficacy against B-cell tumors, thereby highlighting the potential of humanizing amino acid residues in the nanomeric framework region alone to improve both *in vitro* and *in vivo* effectiveness of non-humanized designed nanomaterials. However, following humanization, the prolonged presence of nanomaterials *in vivo* can still elicit the production of autoantibodies against nanomaterials and trigger cytokine release syndrome [171]. In certain clinical applications, the humanization of nano-bodies may be deemed redundant, while non-humanized nano-bodies exhibit relatively short half-lives, thereby eliciting minimal immunogenicity. For instance, clinical trials have utilized non-humanized gallium-68 (68Ga)-labeled anti-erBB-2 (HER2) nanoparticles for positron emission tomography (PET) diagnosis of breast cancer, thereby presenting a challenge in the context of humanized nanoparticle application [176].

The development of CAR has undergone three initial stages. The first-generation CAR comprises the antigen-binding extracellular domain of scFv and the signaling intracellular domain of CD3ζ. Initially, double chimeric receptors were engineered by incorporating the VH and VL chains of immunoglobulins into the constant region of the TCR. Over time, the engineering of CARs has evolved towards a single-chain approach, wherein the antigen-recognition region is constructed using a single-chain antibody. First-generation CARs, comprising an activation domain within the cell, were developed by linking a scFv to the CD3ζ signaling domain of the TCR through a spacer region derived from either the immunoglobulin constant heavy chain or CD8 molecules and anchored with a transmembrane domain. Although first-generation CARs exhibit *in vitro* antitumor activity, their early clinical response has proven disappointing due to poor dissemination, durability, and antitumor efficacy. This may be attributed to the absence of essential costimulatory signals required for physiological activation. The second generation CAR incorporates an intracellular structure with a costimulatory signal domain, comprising an activation domain and a costimulatory domain, building upon the foundation of the first generation CAR. Incorporating costimulatory domains derived from CD28 or 4-1BB (CD137) and OX40 (CD134) enhances the strength of activation signals. Studies have demonstrated that the incorporation of 4-1BB into the CAR architecture can enhance the expansion of CD8^+^ central memory T cells to a greater extent than CD28, resulting in a more persistent anti-tumor response in select patients [177]. In contrast, the third-generation CAR incorporates a combination of CD28 and OX40 (or 4-1BB) costimulatory domains in its intracellular domain, comprising one activation domain and two costimulatory domains. T cells expressing these third-generation CARs demonstrate enhanced activation, effector function, and improved *in vivo* persistence. Furthermore, technical strategies were explored to broaden the repertoire of tumor antigens targeted by CARs. Given their origin from antibodies, scFv molecules possess the ability to specifically bind peptides, carbohydrates, and glycolipids such as Her2, distinct glycoforms of mucin 1 antigen, and disialoganglioside (GD2). These targets are typically recognized by CAR–T cells solely when they are expressed on the cellular membrane. To broaden the repertoire of antigens recognized by scFv-based CARs, encompassing pertinent tumor cell antigens that can be targeted by CAR–T cells, several research groups have successfully engineered single chains specifically targeting HLA-peptide complexes. These TCR-like CARs enable the precise identification of intracellular protein targets. However, clinical experience has demonstrated that TCR-engineered T cells with receptors exhibiting TCR-like specificity may result in off-target toxicity if similar peptide epitopes are expressed on healthy tissues. For instance, modification of TCR affinity against the antigen MAGE-A3 can lead to cardiotoxicity due to the presence of analogous peptide epitopes in titin and neurotoxicity caused by recognition of several members of the MAGE-A protein family that are accidentally expressed in neurons [178]. The scarcity of tumor-specific antigens has been demonstrated as a crucial factor influencing the efficacy of CAR–T therapy. Prior characterization of these antigens is imperative for successful clinical implementation of CAR–T treatment. Regrettably, currently identified solid tumors lack exclusive membrane proteins with specific tumor cell antigenicity.

#### Reconstruction and articulation of armored CAR

Optimal efficacy of early adoptive transfer of tumor-specific T cells in antitumor therapy relies on host preconditioning through cytokine support following total body irradiation, high-dose chemotherapy, and/or cytokine infusion. The optimal clinical response following adoptive T cell therapy exhibited a positive correlation with the intensity of the preceding conditioning regimen. Certain patients were unable to derive benefits from CAR–T therapy due to their inability to tolerate high-intensity conditioning regimens. The blood analysis of a pretreatment preconditioning mouse model revealed a reduction in regulatory CD4^+^ T cells (Tregs) and a significant elevation in serum levels of IL-12 and IFNγ cytokines [137]. Therefore, it can be inferred that implementing additional modifications to enhance the expression of CAR-IL-12 T cells could serve as a promising strategy for optimizing adoptive T cell transfer therapy. It has been demonstrated that the augmentation of CAR–T cells with IL-12 secretion can significantly enhance the functional efficacy of CAR–T cells in non-preconditioned murine models [137]. Given that IL-12 stimulation of T cells induces heightened IFNγ secretion and augmented expression of cytolytic proteins, namely granzyme B and perforin, it effectively counteracts the immunosuppressive effects mediated by TGFβ, thereby resulting in an amplified cytotoxic capacity. The outcomes rely on the body for modifying IL-12 autocrine and T cell secretion of IFN-γ to stimulate CD4 and CD8 T or CAR–T cells subgroup, resulting in the inhibition of Treg-mediated resistance. Therefore, the utilization of gene modification technology to induce autocrine expression of cytokines that enhance T cell functionality is more conducive to immune stimulation and efficacy of CAR–T cells. Studies have demonstrated that, in addition to IL-12, other immune stimulators such as IL-15, IL-7, and IL-18 exhibit the capacity to stimulate cell expansion both *in vivo* and *in vitro*. Furthermore, they are associated with reduced PD-1 receptor expression and cellular mortality. These immune stimulators also play a crucial role in regulating the tumor microenvironment *in vivo* and enhancing the endogenous anti-tumor immune response mediated by T-cells. The second-generation CAR backbone was coupled with an inducible cytokine expression module driven by nuclear factor of activated T cells (NFAT). Following the binding of CAR–T cells to tumor antigens, CAR signaling triggers NFAT phosphorylation, translocation to the nucleus, and activation of NFAT-responsive promoters that facilitate transgene expression. The secreted cytokines IL-7, IL-12, IL-15, or IL-18 not only enhance the survival capacity of CAR–T cells and augment their cytotoxic activity but also attenuate the immunosuppressive tumor microenvironment. The construction of gene-edited armored CAR–T cell structure is currently undergoing diverse optimization processes. The main methods of constructing the armored CAR–T include: expression of single interleukins, expression of two types of interleukins, co-expression of interleukins with other cytokines, expression of interleukin receptors, and expression of interleukin subunits. The CAR–T cells that possess the capability to secrete diverse cytokines are commonly referred to as fourth-generation CAR–T cells (Armored CAR or TRUCK CAR), exhibiting a remarkable potential in modifying the TME and augmenting the activity of CAR–T cells. Currently, a plethora of preclinical studies have substantiated that co-expression of cytokines such as IL-7, IL-8, IL-9, IL-12, IL-15, IL-18, IL-21, and IL-23 can augment the anti-tumor efficacy of CAR–T cells (Table 3) [179].

**Table 3:** Summary of predinical studies on the use of CAR-T cells co-expressing cytokines in the treatment of malignant tumors

Tumor	Targeted antigen	Gene-edited cytokines
Glioblastoma, ovarian cancer and pancreatic cancer	CD70	IL-8andCXCR1orCXCR2 [180]
All or hepatocellular carcinoma	CD19,GPC3	IL-9 [181]
Lung cancer, pancreatic ductal adenocarcinoma	hCD20 Mesothelin	IL-7 and CCL19
prostatic cancer	NKG2D	IL-7
hepatic carcinoma	GPC3	IL-7 and PH20
breast carcinoma	AXL	C7R
Colorectal cancer, pancreatic cancer, stomach cancer CEA	GEA	IL-12
lymphoma	CD19	IL-12
hepatic carcinoma	glypican-3 (GPC3)	IL-12
ovarian cancer	Muc-16	IL-12
leukemia	CD19	IL-15
Cerebral endothelioma	VEGFR-2	IL-15
melanoma	CD19	IL-18
hepatic carcinoma	GPC3	IL-21
chronic lymphocytic leukemia	CD19	IL-21
hepatic carcinoma	GPC3	IL-15 and IL-21
neuroblastoma	GD2	IL-23
prostatic cancer	PSMA	IL-23

The fourth-generation CAR–T, designed to target various immunosuppressive factors in the TME, exhibits additional regulatory functions through gene editing technology. The inhibition of TGF beta factor is achieved through the expression of dominant negative receptor for TGF beta by CAR, leading to enhanced proliferation and persistence of CAR–T cells in tumor models. Armored CAR–T cells exert immune inhibitory control, thereby leveraging immune checkpoints to modulate the survival of cancer cells. For instance, in lymphoma, the presence of immune checkpoint mutations in HVEM/BTLA can enhance lymphoid stroma activation and facilitate recruitment of follicular helper T cells to the TME. To counteract this effect, engineered CAR–T cells can be designed to secrete the extracellular domain of HVEM combined with BTLA structure, thereby inhibiting tumor cell growth and impeding TME development [182]. CAR–T cells engineered to secrete anti-PD-L1 antibodies effectively mitigate T-cell exhaustion and facilitate recruitment of NK cells to the tumor microenvironment [183]. Moreover, low oxygen metabolism enhances the TME’s glycolysis and elevates lactic acid production, thereby facilitating tumor cell proliferation while concurrently suppressing the functionality and survival of T and NK cells. Consequently, armored CAR–T cells secrete catalase (CAR-CAT) to effectively counteract the hypoxic and reactive oxygen species (ROS)-rich TME. To overcome these barriers, an alternative approach is to genetically modify the CAR to express antioxidant factors, such as n-acetyl cysteine (NAC), which can mitigate DNA damage in CAR–T cells and attenuate CAR–T cell activation-induced cell death, thereby enhancing their anti-tumor efficacy [184]. The fourth-generation CAR-driven cytokines also facilitate the elimination of antigen-negative tumor cells by bystander T cells.

In summary, we postulate that genetically modified armored CAR–T cells hold promise for targeted eradication of heterogeneous tumor cells and their microenvironment in clinical translational research, particularly through the development of multi-interleukin (IL) and interleukin combined with other cytokine gene-edited CAR–T cells. Re-targeting engineered T cells can enhance the survival and proliferation of CAR–T cells through autocrine or paracrine cytokines, leading to bystander cells playing an anti-tumor role, improving the TME, inhibiting tumor growth, altering immune cell chemotaxis patterns, and suppressing angiogenesis. This may represent a crucial avenue for conquering solid tumors in the future.

#### CAR–T cells with an inverted cytokine receptor (ICR)

The immunosuppressive microenvironment of solid tumors, characterized by nutrient and oxygen deprivation, the presence of inhibitory cells and cytokines such as IL-4, vascular abnormalities, and endothelial dysfunction, represents significant barriers to effective cancer immunotherapy. Enhanced T cell functionality within a suppressive microenvironment is achieved by incorporating inverted cytokine receptors (ICRs), wherein the extracellular domain of an immunosuppressive interleukin receptor (e.g., IL-4) is fused with the intracellular domain of an immunostimulatory interleukin receptor (e.g., IL-7). This conversion of IL-4 signaling inhibition into a signal that amplifies the antitumor efficacy of CAR–T cells at the tumor site (signal 3-stimulatory cytokine signaling) serves to safeguard CAR–T cells against cytokine-mediated suppression. The so-called ICRs have been extensively investigated for their ability to modulate specific immunosuppressive cytokines within the TME, thereby enhancing the efficacy of CAR–T cell therapy. For instance, the IL-4/IL-7 ICR (4/7ICR) exhibits an affinity towards immunosuppressive IL-4, while effectively converting downstream signals into immunostimulatory IL-7 receptor signals. The involvement of IL-4 enabled the preservation of Th1 phenotype and cell viability *in vitro* for CAR–T cells with a 4/7 ICR, while maintaining the antigen specificity of the CAR and exhibiting sustained potent antitumor activity *in vivo*. The utilization of this technique represents a strategic approach to enhance the antitumor efficacy of CAR-modified T cells in the presence of immunosuppressive cytokines. Given the pivotal role of the pleiotropic cytokine IL-21 in regulating CD8^+^ T cell effector function and naive CD4^+^ T helper (Th) cell polarization, we engineered 4/21 ICR-CAR–T cells in the presence of IL-4, resulting in their differentiation into a Th17-like phenotype and subsequent rapid tumor eradication [185]. The present study suggests that distinct mechanisms may underlie the promotion of antitumor activity between the 4/7 ICR and the 4/21 ICR. Mechanistic studies revealed that both 4/7 and 4/21 ICRs were capable of initiating phosphorylation-STAT signaling cascades; however, activation of the 4/7 ICR resulted in STAT5 phosphorylation, whereas the 4/21 ICR exhibited a higher propensity for inducing STAT3 phosphorylation. The activation of STAT3 is widely recognized as a pivotal factor influencing the efficacy of CAR–T cell therapy [135]. As T cell effector Granzyme B serves as the target gene expression molecule of IL-21 signaling, 4/21 ICR-CAR–T cells exhibited more robust and sustained cytotoxicity compared to 4/7 ICR [186].

By utilizing the signal transduction effect of ICR receptors, inhibitory factors present in the tumor microenvironment can be converted into intracellular excitatory signals to promote the survival and proliferation of CAR–T cells. The feasibility of genetically modified T cells thriving in a suppressive tumor environment has been demonstrated. Additionally, the researchers emphasized that providing transgenic T cells with activation signals for antigen recognition (signal 1), costimulation (signal 2), and cytokine support (signal 3) that mimic the physiological TCR or CAR is crucial for enhancing the persistence and memory formation of transgenic T cells *in vivo*, thus significantly extending the tolerance of CAR–T cells to inhibitory environments. This approach resulted in improved *in vivo* survival and enhanced anti-tumor effects. Conversely, exploring the potential of inhibiting CAR–T cell function through ICR technology in a negative feedback manner warrants equal investigation. In the context of cytokine activation, these negative feedback CAR–T cells have the ability to convert a portion of activating signals into inhibitory signals, thereby potentially serving a dual role in not only mitigating CRS and ICANS but also alleviating CAR–T cell exhaustion. This holds significant research value for potential clinical applications.

#### CAR–T therapy targeting multiple surface antigens on tumor cells

Target antigen loss constitutes a well-established mechanism of immune evasion by tumors and represents the primary cause for disease recurrence. A strategy utilizing CAR–T cells targeting multiple antigens may potentially overcome current limitations and enhance therapeutic efficacy. Hegde et al. utilized multi-color flow cytometry and immunofluorescence techniques to investigate the single-cell co-expression patterns of HER2, IL-13Ra2, and EphA2 in primary GBM samples. Through mathematical modeling analysis, they demonstrated that simultaneous targeting of HER2 and IL-13Ra2 optimizes the therapeutic efficacy of T-cell products [187]. Reinforced targeting of these tumor-associated antigens can effectively counteract the antigen escape mechanism. By generating multiple cell populations expressing distinct chimeric antigen receptors and co-delivering them either simultaneously or sequentially, it is possible to target more than one antigenic structure, thereby enhancing the elimination of tumor cells and reducing heterogeneous tumor burden. Multiple strategies can be employed to effectively target a diverse range of antigens using CAR–T cell therapy. Firstly, the administration of an engineered T-cell population and a unique CAR could be considered either sequentially or concurrently. One of the challenges associated with this approach is the necessity for conducting multiple production runs in order to generate a single CAR population, which can be both an inefficient and costly process. Given the multifactorial impact on *in vivo* persistence of CAR–T cells, including the immunogenicity of the CAR structure, composition of the costimulatory domain, and cell source, which collectively influence clinical efficacy, sequential administration emerges as a viable strategy to sustain or extend CAR cell concentration levels *in vivo*. By considering the decay kinetics of CAR–T cells within an organism, infusion can be repeated in cycles and targeted towards specific cell subsets. Clinical research findings further support the adoption of sequential administration for effective implementation of CAR–T cell therapy [188]. The reported findings demonstrate that sequential infusion of CAR–T cells targeting the same or distinct antigen receptors can augment the anti-tumor efficacy, mitigate tumor drug resistance, and enhance the clinical remission rate. The clinical findings indirectly validate the potential clinical applicability of designing CAR constructs targeting multiple antigens at the single-cell level. The multi-antigen targeting CAR cells can be classified into three distinct types. 1) Encoding bicistronic vectors that carry two different CARs in the same cell. 2) The T cells were simultaneously engineered using two distinct CAR constructs (cotransduction) to generate three subsets of CAR–T cells, encompassing both dual and single CAR-expressing cells. 3) Alternatively, a bispecific CAR or a tandem CAR, such as a CD19-CD20 tandem CAR, can be employed to represent both CARs on the same chimeric protein using only one vector [189]. Schneider et al. constructed CD19 and CD20 (or CD22) as distal receptor expression constructs on the CAR protein, which were subsequently compared with single-antigen CARs. The findings demonstrated that tandem CAR–T and single-antigen targeted CAR–T exhibited comparable or superior efficacy compared to single-antigen targeted CAR constructs in conventional disease models, while tandem CAR–T displayed enhanced effectiveness and reduced toxicity in high disease burden settings [189]. Multiantigen-targeted CAR therapies have been assessed in several early-phase clinical trials, demonstrating their potential for targeted immunotherapy. In a phase 1 trial, Cordoba et al. demonstrated promising safety and efficacy of AUTO3 (autologous transduced T cells expressing anti-CD19 and anti-CD22 CARs) [190]. No instances of dose-limiting toxicity, AUTO3-related severe cytokine release syndrome, or neurotoxicity have been documented. The response rate at the one-month follow-up after treatment was 86% (13 out of 15 patients). The 1-year overall and event-free survival rates were recorded at 60% and 32%, respectively. The analysis of relapse cases suggests that the limited long-term persistence of AUTO3 may contribute to its occurrence, highlighting the need for enhancing CAR–T cell persistence. In particular, further exploration is required to investigate the clinical application of sequential multi-course infusion as a potential strategy to reduce recurrence rates. Tong et al. employed TanCAR7, a dual antigen-targeting tandem CAR–T cell therapy against CD19 and CD20, in an open-label, single-arm phase I/2a trial (NCT03097770) to evaluate its efficacy in 33 patients with relapsed/refractory non-Hodgkin lymphoma (R/R NHL). The study reported an overall response rate of 79% (95% confidence interval (CI), 60% to 92%), with a complete response rate of 71%. The 12-month progression-free survival rate was determined to be 64% (95% CI, 43% to 79%) based on our analysis. In the present study, TanCAR7 T cells demonstrated robust and enduring antitumor responses in patients with R/R NHL without experiencing grade 3 or higher cytokine release syndrome (CRES) [191]. By targeting B cell maturation antigen (TNFRSF17; BCMA) and G protein-coupled receptor class C group 5 member D (GPRC5D), the prevention of BCMA escape-mediated relapse in MM models can be achieved [192]. Subtherapeutic doses of various forms of dual-targeted cell therapy were compared in a breast cancer model (I. Parallel generation and hybrid single-targeted CAR–T cells; II. Bicistronic constructs expressing different CARs from a single vector; and III. Dual scFv ‘single-stem’ CAR design). The bicistron and combined approaches demonstrated superior efficacy in targeting BCMA-negative breast cancers, while the bicistron approach outperformed the combined approach in breast cancers expressing double antigens. Mechanistically, it is hypothesized that the co-expression of both CARs on individual cells augments the potency of CAR–T cell/target cell interaction.

The combinatorial optimization design of dual (or multiple) antigen-targeting CARs is crucial in the context of heterogeneous tumors and has a significant impact on clinical outcomes. The constitutive expression intensity of different tumor-associated antigens varies across the same or different types of tumors, and no definitive principles exist to guide its determination. The absence of universally applicable characteristics for patient treatment and the necessity for personalized analysis impede the potential industrial production of multi-antigen targeted CAR–T cells, significantly augment the economic burden on patients, and engender resource wastage. Given this characteristic, the investigation of universal CAR–T cells also represents a focal point for future development.

#### Designing switchable domain universal CAR–T cells

To ensure flexibility in target selection, various groups have been actively exploring strategies to develop universal chimeric receptors, thereby effectively addressing the trade-off between efficacy and safety through the segregation of functional domains within the CAR structure. In principle, the universal CAR structure (UniCAR) domain is tethered to tumor surfaces through bidirectional mediation of known tag domains (tag molecules or targeting modules (TM)), thereby activating CAR–T cells and inducing tumor-specific cytotoxic effects. On one hand, these tag molecules are engineered to specifically target TAA domains by incorporating TAA-specific scFv structures, nanobodies (NB), immunoglobulin G (IgG), and small peptide molecules (PET tracers). These soluble mediators are designed for *in vivo* injection and subsequent redirection towards the tumor site. On the contrary, the tag molecule possesses a universally recognized antigen recognition domain. Only when this domain recognizes and binds to the corresponding universal CAR, thereby activating CAR–T cells, can it elicit tumor immunotoxic effects. Therefore, the extracellular binding domain of the universal CAR does not recognize TAA on tumor cells; instead, it specifically recognizes epitopes on the tag molecule for labeling. The modified generic CAR–T cells themselves exhibit a state of inactivity and indolence, while the mediated tumor killing occurs in a dose-dependent manner with respect to the tag-molecule, thereby enabling reactivation of CAR–T or alteration of the targeting protein at any given time by manipulating the supply of tag-molecules. Consequently, this allows for effective targeting of multiple TAAs through CAR–T cells expressing a single receptor, thus achieving therapeutic efficacy against multi-targeted antigens. To ensure the specificity of universal CAR recognition and minimize on-target/non-tumor effects, aptamers devoid of membrane surface presence on normal human cells can be selectively chosen for these universally applicable CAR receptor structures. The potential binding partners identified include biotin-avidin, Fc receptor CD16 and antibodies, peptide neoepitopes (PNE) and corresponding anti-peptide epitope single-chain antibodies, as well as fluorescein and anti-fluorescein single-chain antibodies. The tag-molecule-universal CAR strategy enables the concurrent targeting of multiple antigens, facilitating dynamic modification of the target repertoire during treatment and providing flexibility in adjusting the targeting specificity and activity of CAR–T cells through *in vivo* redirection of tag molecules and modulation by switch molecules. The success of this strategy is contingent upon a two-component system, thereby necessitating consideration of the bioavailability, biodistribution, and metabolic half-life of both the soluble tag molecule and chimeric receptor universal T cells. The metabolic profile of the signature molecules plays a crucial role in regulating both the efficacy and adverse effects of universal CAR–T therapy, thereby determining its persistence. For instance, the transient removal of tag molecules (scFvs or NB) *in vivo* can promptly deactivate universal CAR–T cells [193], thereby expeditiously mitigating adverse reactions and facilitating antigen-specific targeting [193]. Following the eradication of tumor cells, signature molecules with prolonged metabolic cycles can be employed for *in vivo* immune surveillance, obviating the need for frequent infusions. Lohmueller et al employed a combination of a biotinylated tumor-specific antibody and a monomeric streptavidin 2 (mSA2) biotin-binding domain CAR–T (mSA2-CAR–T). The mSA2 CAR exhibits versatility in binding to a biotinylated tumor-specific antibody, thereby potentially enabling targeting across diverse tumor types [194]. mSA2 CAR–T cells can mediate cancer cell lysis and induce IFNγ production in a dose-dependent manner through biotin antibody targeting. Pishali et al. employed a preclinical model of prostate cancer thatexpressed prostate stem cell antigen (PSCA) to demonstrate the efficacy of the UniCAR platform in targeting solid tumors both *in vitro* and *in vivo* [195]. In the low tumor load and high load model, transplantation of UniCAR–T cells targeting module resulted in a significant delay in tumor growth and prolonged survival time of mice with immune-defective tumors. The activation of uniCAR–T cells was found to concurrently induce the up-regulation of immunosuppressive molecules, such as programmed death ligand, thereby establishing a link between its activity and the adaptive immune escape response. This observation sheds light on the potential immune evasion mechanism associated with the UniCAR platform. Switchable domain-uniCAR–T have not yet undergone human testing, thus the translation of their unfixed specificity and modular design into enhanced antitumor efficacy remains to be determined.

### Reengineering strategies to ameliorate adverse effects of CAR–T therapy

Controlling traditional CAR–T cells as a viable drug poses challenges, given their propensity for uncontrolled proliferation that can unpredictably exacerbate side effects associated with CAR–T cell therapy, such as cytokine release syndrome, targeted/non-tumor toxicity, or neurotoxicity. The untreated mismanagement of these adverse effects may lead to fatality. Considering the adverse events and primary bottleneck associated with CAR–T cell therapy, it is imperative to develop pharmacological methods for *in vivo* control. The safety and effectiveness of various methods have been extensively investigated in previous studies. These studies primarily encompass genetic-level T-cell modification and reconstitution in the context of CAR–T cell therapy. For instance, the implementation of a suicide mechanism in CAR–T cells offers a secure and effective control strategy. Amatya et al. developed a CAR construct incorporating a CD28 co-stimulatory domain and a suicide gene, alongside the lymphocyte activation molecule F7 (SLAMF7) [196]. The robust expression of SLAM F7 on the surface of multiple myeloma (MM) cells, while being absent on normal non-hematopoietic cells, renders it a promising target for CAR–T cell therapy in MM. The suicide gene encodes a dimeric domain that interacts with the Caspase-9 domain [196]. Researchers demonstrated the *in vitro* expression of SLAMF7 with suicide gene-specific CAR in T cells, as well as the specific recognition and eradication of SLAMF7-positive cells in mice tumor models. In contrast, the administration of dimeric AP1903 allows for targeted elimination of these genetically modified T cells when necessary [196]. The dimeric drug effectively eradicated 90% of the engineered cells within a span of 30 min following a single administration to the patient. In addition, the “graft-*vs*-host” disease associated with CAR–T adverse reactions was effectively managed in the absence of tumor recurrence [197]. However, suicide strategies primarily lead to the complete elimination of CAR–T cells, potentially resulting in premature termination of the intervention. Therefore, it is imperative to exert control over the expansion of non-lethal CAR–T cells in order to enhance the efficacy and safety profile of CAR–T cell therapy [198]. Wu et al. proposed an alternative strategy that harnesses the benefits of chemically induced dimerization tools, employing a designed on-switch to achieve tunable CAR–T cell activity *in vivo* through exogenous administration of a small-molecule activator [199]. The CAR peptides were fractionated to ensure the segregation of recognition and signaling domains onto distinct peptide fragments. The application of chemically induced dimerization tools facilitated the proximity of the two peptides, thereby reconstructing the CAR in a supramolecular architecture rather than a macromolecular structure. Receptor activation alone does not suffice to initiate signal transduction, nor does the addition of a protein dimerization agent. The two signals, namely “receptor activation” and “protein dimerization,” synergistically orchestrate CAR reconstruction, thereby establishing an “AND” logic gate. Moreover, this approach enhances the robustness and durability of CAR both *in vitro* and *in vivo*, thereby enabling precise regulation of CAR function without compromising its cellular integrity.

Roberto provides a simpler solution by adjusting the CAR, such as the scFv structure domain with enhanced binding affinity, to address the complex and challenging integration of genetically modified (GM) genomes. This is because optimizing receptor affinity threshold leads to a more pronounced T cell response effect [200]. The efficacy of CAR–T cell therapy cannot be enhanced by high antigen affinity; however, it is more likely to specifically bind to low-expressed target antigens in normal cells or tissues and induce immune adverse reactions. The authors also established T-cell platforms for the presentation of CAR function through antigen binding and/or signal-based screening. The experimental demonstration of the value of functional signal-based screening in regulating CARs establishes its potential as a universal strategy for designing CARs with enhanced antigen specificity and target cell selectivity. But the concept of the product may be influenced by numerous uncertain factors, necessitating further validation through clinical research.

The comprehensive CAR–T adverse reaction management plan may also encompass additional strategies. The present investigation incorporates CAR-based small molecule switchable T cell receptors, Synthetic fission receptors, combined target antigen recognition, Notch receptor synthesis, Bispecific T-cell adhesion receptors, and dual inhibitory chimeric antigen receptors (iCAR). These strategies also represent effective approaches for enhancing the safety of engineered T cells, as outlined in the following presentation.

#### The development of bioorthogonal targeted CAR–T cells in a synthetic setting

The interaction between an antigen and its homologous receptor or antibody specificity plays a crucial role in numerous biomedical applications. The highest degree of specificity is achieved when the antigen is a non-natural (synthetic) molecule that has been introduced into the biological environment as a bioorthogonal ligand. The binding of synthetic ligands to cognate antibodies is believed to be orthogonal to all other recognition events *in vivo*, thus conferring a high degree of specificity. It has been observed that intimate cellular interactions between immune cells and tumor cells play a pivotal role in intercellular immune recognition, communication, activation, and cytotoxicity during anti-tumor immune responses. The strength of cell-cell interactions can be enhanced through the interaction between cell surface adhesion molecules and receptors, thereby increasing avidity [201]. Artificial modification of ligand receptors on the T cell surface may serve as a simple and effective strategy to enhance the anti-tumor efficacy. The incorporation of highly potent and precise synthetic targets can enhance the therapeutic efficacy of immune cells against tumors and minimize unnecessary toxicity in cancer treatment. Ma et al. utilized the lymphoid tropism of adiponectin haptens to conjugate a fluorescein ligand (haptens) onto the surface of antigen-presenting cells *in vivo*, thereby serving as an efficacious ‘vaccine’ for targeted activation of specific CAR–T cells [202]. These CAR–T cells are directed to proliferate within the immunologically naive microenvironment of antigen-presenting cells, leading to a substantial expansion in the population of CAR–T cells and successful targeted elimination of tumor antigens. CAR–T cell therapy alone exhibited limited efficacy against tumor growth, whereas repeated administration of the fluorescein vaccine demonstrated a significant inhibitory effect on tumor progression [202]. An alternative approach involves modulating cell surface carbohydrates by incorporating non-natural sugars through bioorthogonal sugar metabolic engineering, which has emerged as a potent technique for artificially labeling and modifying target cells [203]. This technology exemplifies the targeting principle of artificial bioorthogonality by facilitating a chemical reaction between functional chemical reporter molecules and their complementary groups *in vivo*. Pan et al. demonstrated that by employing Ac4GalNAz and Ac4ManNBCN for sugar metabolism engineering, the paired chemical reporter group (-N3/-BCN) could be introduced into CAR–T cells and tumor cells, respectively. This bioorthogonal chemically guided specific targeting approach facilitates the connection and activation of CAR–T cells towards tumors, enhancing their interaction through bioorthogonal click chemistry on functional groups present on the cell surface. Consequently, this strategy improves the specific recognition, migration, and selective anti-tumor effect of CAR–T cells [204]. The artificial bioorthogonal targeting strategy significantly enhanced the accumulation, deep tissue penetration, and tumor homing of CAR–T cells in comparison to unlabeled cells. The incorporation of this approach significantly enhances the selective recognition and anti-tumor efficacy of CAR–T cells both *in vitro* and *in vivo*, thereby presenting substantial potential for effective clinical cellular immunotherapy while mitigating on-target/non-tumor adverse events in patients. Although bioorthogonal targeted CAR–T presents a promising technical strategy for the clinical treatment of tumors, the intricate production process and high manufacturing costs also impede its large-scale production, necessitating further exploration of more cost-effective optimization schemes.

#### CAR–T cells engineered through mRNA-based gene modification technology

Retroviral vectors and slow virus transduction represent the conventional approach for genetically modifying T cells with CARs. However, the stability of transgene expression can be problematic when unexpected cross-reactivity of transgenic immune receptors leads to severe adverse reactions. The integration of genetic problems poses a permanent risk of uncontrolled immune response, as evidenced by the continuous expression of CARs. Alternatively, the concern regarding lentivirus or retrovirus-mediated introduction of CARs into CTLs also encompasses the well-established risk of insertional mutagenesis leading to malignant transformation. The management of these risks typically necessitates drug intervention and poses challenges in terms of control, exhibiting a lack of self-limitation. The transient and self-limited expression pattern of CAR structure enables controlled cytotoxicity within a defined timeframe, thereby effectively managing the clinical adverse reactions associated with CAR–T therapy and establishing it as a safer approach. Notably, mRNA-based TCR and CAR transfection (IVT mRNA) presents a favorable option for gene transfer due to its lack of integration-related safety concerns. This method can serve as a standard approach for ensuring safety and screening for adverse reactions. If the tested receptors exhibit no undesirable off-target or de-tissue reactivity, they may be retrovirally transduced for long-term efficacy and safety. The fulfillment of this requirement can be achieved through electroporation of T cells that have been genetically modified with mRNA encoding CARs (mRNA-EP) [205]. The technique of RNA electroporation is a well-established and readily applicable method in the field of cell engineering with its efficacy having been validated through clinical studies [206]. The activation and proliferation of T cells following receptor transfer can result in dilution and eventual loss of the transferred receptor, thereby limiting the duration of CAR expression. This transient CAR expression restricts the timeframe for T cells to exhibit their antitumor activity, necessitating repeated administrations of CAR-modified T cells. Consequently, a substantial quantity of modified T cells is imperative for clinical applications. This significantly surpasses the quantity of cells obtained from cancer patients through leukapheresis. Therefore, prior to RNA transfection, it is imperative to ensure T-cell expansion. However, prolonged and vigorous proliferation followed by repeated stimulation often leads to the induction of T cell senescence, thereby impeding the functional capacity of T cells. Therefore, the selection of an appropriate T-cell expansion cycle assumes paramount importance. Krug et al. demonstrated that a modest expansion of T-cells can be achieved within a relatively short timeframe (2 weeks), thereby mitigating excessive progression towards late-stage T-cell differentiation [205]. The functional cycle of CAR–T cells is contingent upon the expression cycle of the CAR, and the observed reduction in CD62L expression subsequent to mRNA electroporation suggests a differentiation towards effector phases, such as effector memory T cells and effector T cells. Although transient in a patient post-transplantation, CAR–T cells only require demonstrable cytotoxicity towards target cells. Ensuring adequate cytotoxicity during the transient expression of CAR is advantageous for enhancing anti-tumor efficacy and mitigating on-target/non-tumor effects [207]. The study conducted by Yoon and colleagues demonstrated that adoptive transfer of Her2/neu RNA CAR electroporated T cells in a SKOV3 xenograft model resulted in reduced tumor growth rates compared to the transfer of mock-transfected T cells [208]. The anti-cancer efficacy of IVT mRNA CAR–T cells targeting the regulatory cell growth protein FRα was demonstrated by Schutsky et al. in an ovarian cancer model [209]. In the *in vitro* system, human FRα-targeted IVT mRNA CAR–T cells exhibited cytotoxicity against human ovarian cancer cell lines OVCAR3, A187, and SKOV3. Moreover, the utilization of FR alpha target mRNA CAR–T exhibits a remarkable capacity to impede tumor cell proliferation in an experimental mouse model of cancer. Furthermore, preclinical studies have demonstrated the remarkable therapeutic efficacy of IVT mRNA CAR–T in various malignant tumors including mesothelioma, breast cancer, neuroblastoma, glioblastoma multiforme, and melanoma, with no observed adverse reactions. In a clinical trial, Svoboda et al. employed mRNA-engineered T cells targeting CD19 for the treatment of patients diagnosed with relapsed or refractory classical Hodgkin lymphoma (CHL) [210]. After the administration of CD19 CAR–T cells, transient responses were observed in four patients. A complete response was achieved by one patient, a partial response was achieved by another patient, while two patients exhibited no response. The transient expression of the inserted CAR mRNA in T cells did not induce significant cytotoxicity in any of these cells. In a phase 0 clinical trial, Tchou et al. reported the absence of any observed adverse effects in patients with metastatic breast cancer who received intratumoral c-Met IVT mRNA CAR–T cells [211]. However, immunohistochemical analysis of resected tumors revealed a significant extent of necrosis, indicating that c-Met IVT mRNA CAR–T cells exhibit potent anticancer effects in patients with breast cancer. During a phase I clinical trial investigating the efficacy of a CAR–T targeting mesothelin mRNA in patients diagnosed with malignant pleural mesothelioma, an adverse event was observed in one participant, characterized by a sudden elevation in immunoglobulin G (IgG) levels following the second administration [168]. Anaphylaxis can also result from IgE-mediated immune responses, wherein exogenous CAR fragments induce mast cell degranulation. Administration of multiple doses of CAR–T cell infusion may potentially contribute to anaphylaxis; however, modifying the dosing regimen has been shown to mitigate the occurrence of subsequent anaphylactic reactions [168]. The fully humanized modified CAR structure may potentially contribute to the amelioration of allergic reactions, necessitating further validation in subsequent investigations. According to current research data, IVT mRNA CAR–T cell therapy has demonstrated a favorable response in both preclinical and clinical studies for hematological disorders as well as solid tumors. The construction technology of this treatment platform still holds great potential for optimization. As more and more clinical research data are published, its clinical application value will be better demonstrated.

#### CRISPR/Cas9-mediated elimination of endogenous αβT cell receptors

TCRs possess an inherent capacity to elicit responses towards non-autologous tissues and exhibit the ability to recognize allogeneic human leukocyte antigen (HLA) molecules as well as other minor antigens. The adoptive transfer of donor lymphocytes with endogenous αβTCR can lead to graft-*vs*-host disease (GVHD) by identifying HLA mismatching recipients’ allogeneic antigens, while the presence of foreign HLA molecules on donor T cells may result in rejection. Therefore, it is hypothesized that the presence of a double defect in the donor cell TCR and HLA-I T cells may lead to a reduction in alloreactivity, thereby minimizing the likelihood of causing GVHD. Given that β2-microglobulin (β2M) serves as a crucial subunit of HLA-I protein, depletion of β2M effectively circumvents rapid depletion of allogeneic T cells expressing foreign HLA-I molecules. In order to address GVHD and enhance the persistence and efficacy of CAR–T therapy, a genome editing strategy can be employed to disrupt the expression of TCR and β2M genes in CAR–T cells. This approach enables the generation of allogeneic universal CAR–T cells, thereby reducing clinical adverse events while improving their *in vivo* durability and therapeutic effectiveness. The CRISPR/Cas9 system possesses a distinctive capability to simultaneously edit multiple loci, rendering it suitable for concurrent modulation of multiple genes. Consequently, CRISPR-Cas9 holds the potential to achieve loss of function (LOF) in any genetic or epigenetic target. CRISPR/Cas9-mediated depletion of endogenous TCR gene *TRAC*, encompassing both alpha and beta chains, has exhibited remarkable efficacy in preclinical investigations, employing gene editing to suppress TCR expression. By eliminating the *TRAC* gene continuous region, this intervention enhances the adaptability of CAR–T cells, presenting a valuable opportunity for their therapeutic application. Eyquem et al. employed CRISPR/Cas9 to integrate CAR genes into the *TRAC* site while simultaneously excising the TCR gene. In a mouse model of ALL, normal CAR expression was observed in T cells, while TCR-deficient CD19-specific CAR–T cells exhibited increased T cell potency and reduced terminal differentiation and exhaustion. These findings of such modified CAR–T cells may stimulate more effective ant-T cells [212]. Therefore, the removal of *TRAC* or β2 M has been demonstrated to enhance the persistence of CAR–T cells *in vivo* and facilitate their generation from allogeneic donor T cells. Moreover, the combination of multiple genome editing technologies and simultaneous disruption of certain gene loci that inhibit T cell immune function, such as immune checkpoint receptors, TGF-β receptor II (TGFBR2), and GM-CSF, can further augment the survival capacity, clinical efficacy, and safety profile of CAR–T cells. The safety of multiple CRISPR-Cas9 editing of the human genome is further validated by a clinical study [213]. In conclusion, the advancements in genome editing have significantly broadened the potential of CAR–T cell-based adoptive cell therapy. Based on a more comprehensive study, the application of CRISPR-Cas9 can enhance the safety and efficacy of CAR–T cells while also improving their accessibility, thus leading to optimal clinical outcomes.

#### Application of dual CAR or inhibitory CAR (iCAR)

The main challenge in cancer treatment lies in accurately distinguishing between tumor cells and normal cells. In order to enhance the tumor-specificity of CAR–T cells and mitigate the adverse effects associated with “on-target, off-tumor” recognition, various dual CAR constructs were designed to augment the specificity of CAR-based tumor therapy by exploiting either the coexistence of multiple TAAs on malignant cells or the absence of normal cell antigen proteins due to common genetic material loss or loss of heterozygosity (LOH). The proposed treatment concept effectively tackles the fundamental challenge of distinguishing tumor cells from normal cells, making it potentially applicable to a wide range of tumors. One strategy involves segregating the primary activation of T cells from costimulatory signaling events through the utilization of individually expressed CARs targeting distinct antigens. The split T-cell activation signal allows for tumor specificity, as dual CAR-transduced T cells can only achieve full activation when both CARs are targeted to tumor cells, rather than recognizing a single antigen in healthy tissue. Chen et al. employed two different scFv fusions to separately target the 4-1BB structure domain and CD3 ζ domain structure, enabling simultaneous identification of two antigens and achieving full activation of T cells [214]. Similarly, the identical outcomes were achieved utilizing the “And gate” approach, wherein the logical regulation of CAR–T cell response necessitates dual antigen binding. Fisher et al. focused on GD2-expressing neuroblastoma and utilized the well-established risk-sensing specificity of γδTCR to engineer a Vγ9Vδ2^+^ CAR–T cell, incorporating separate receptors for T cell activation signals 1 and 2 [215]. Specifically, Vγ9Vδ2^+^ T cells were engineered to express an anti-GD2 CAR harboring a costimulatory signal-independent intracellular domain derived from the NKG2D adapter protein DAP10. The CAR construct provides the second requisite signal for T-cell activation, while the Vγ9Vδ2^+^ TCR supplies the initial signal necessary for T-cell activation. The design ingeniously exploits the sensing ability of Vγ9Vδ2^+^ TCR cells, the predominant subset of γδTCR, to detect tumor cell risk while lacking cell activation characteristics. By supplementing with DAP10, it provides a costimulatory signal for effective T cell activation and cytotoxicity, thereby obviating the need for two distinct tumor-associated antigens. In the killing assay, GD2-expressing neuroblastoma cells co-cultured with Vγ9Vδ2 TCR-T cells exhibited efficient lysis, while neuroblastoma cells expressing GD2 but not co-cultured with Vγ9Vδ2 TCR-T cells remained unaffected. The distinction between X-on and X-off tumors presents the potential for enhanced safety in immunotherapy and an expanded repertoire of therapeutic targets. Although these strategies can mitigate the occurrence of ‘on-target off-tumor’ effects, their applicability is not universal and implementation remains challenging. An alternative approach to enhance the efficacy and safety of CAR–T cell therapy involves the utilization of iCAR, thereby presenting a dual CAR–T cell strategy for tumor treatment [216]. Given that LOH leads to an irreversible depletion of genetic material, targeting tumors exhibiting LOH-associated allelic loss offers a promising avenue for selective therapeutic interventions. The etiology of the majority of LOH cases remains elusive. The occurrence of loss of heterozygosity (LOH), as observed in tumor suppressor genes exhibiting high-frequency LOH, is likely to be influenced by the selection of the malignant phenotype. However, the majority of mutations are likely incidental outcomes resulting from random loss and genetic drift, thus being classified as “passenger mutations” upon entering the initial tumor clone. The HLA protein cluster represents the predominant tumor in the population with LOH alleles, making it a preferred target for blockade. Exploiting the immune evasion mechanism characterized by the loss or down-regulation of human leukocyte antigen DR (HLA-DR) in a significant proportion of hematologic malignancies, Fei et al. devised a CD28/CD3-based anti-CD19 CAR and simultaneously expressed an intracellular PD-1 inhibitory construct-based anti-HLA-DR iCAR on the same cells, resulting in dual CAR-NK cells. The dual CAR-NK cells exhibit a preferential targeting towards CD19^+^HLA-DR^neg^ cells over CD19^+^HLA-DR^+^ cells, while the presence of surrounding HLA-DR-expressing cells does not impact the targeting selectivity of dual CAR-NK cells [217]. In a murine tumor model, HLA-DR positive cells exhibited resistance to dual CAR-NK cell-mediated cytotoxicity *in vivo*. The inhibition mediated by Icar was found to exhibit a positive correlation with the density of iCAR and HLA-DR. The iCAR platform, therefore, holds the potential to enhance the safety of CAR cell therapy. Following the principle that killer cell immunoglobulin-like receptors (KIR) interact with HLA ligands expressed on the surface of normal cells, thereby inhibiting NK cell activation, Tao et al. engineered a chimeric construct by fusing the extracellular domain of KIR 2DL2 with the intracellular domain of PD-1, resulting in the development of an innovative inhibitory CAR termed KIR/PD-1-based CAR (iKP CAR) [218]. The expression of PD-1 in T cell activation regulates the inhibitory proteins, thereby controlling excessive T cell activation. Therefore, iKP CAR effectively suppresses CD19 CAR activation signals via the PD-1 domain, and *in vitro* experiments demonstrate that CD19-CAR–T cells carrying iKP cars (IKP-19-CAR–T) exhibit robust cytotoxicity. Furthermore, in a xenograft model of CD19^+^ HLA-C1^-^Burkitt lymphoma, IKP-19-CAR–T cells display comparable anti-tumor activity to that of CD19-CAR–T cells. In both *in vitro* and xenotransplantation models, CD19^+^ HLA-C1^+^ B cells from healthy individuals were found to be preserved. Therefore, it is speculated that the utilization of “iKP-19-CAR–T” holds significant promise as a viable strategy for mitigating B cell aplasia induced by “CD19-CAR–T” cells in clinical therapeutic interventions.

**Figure 2:**
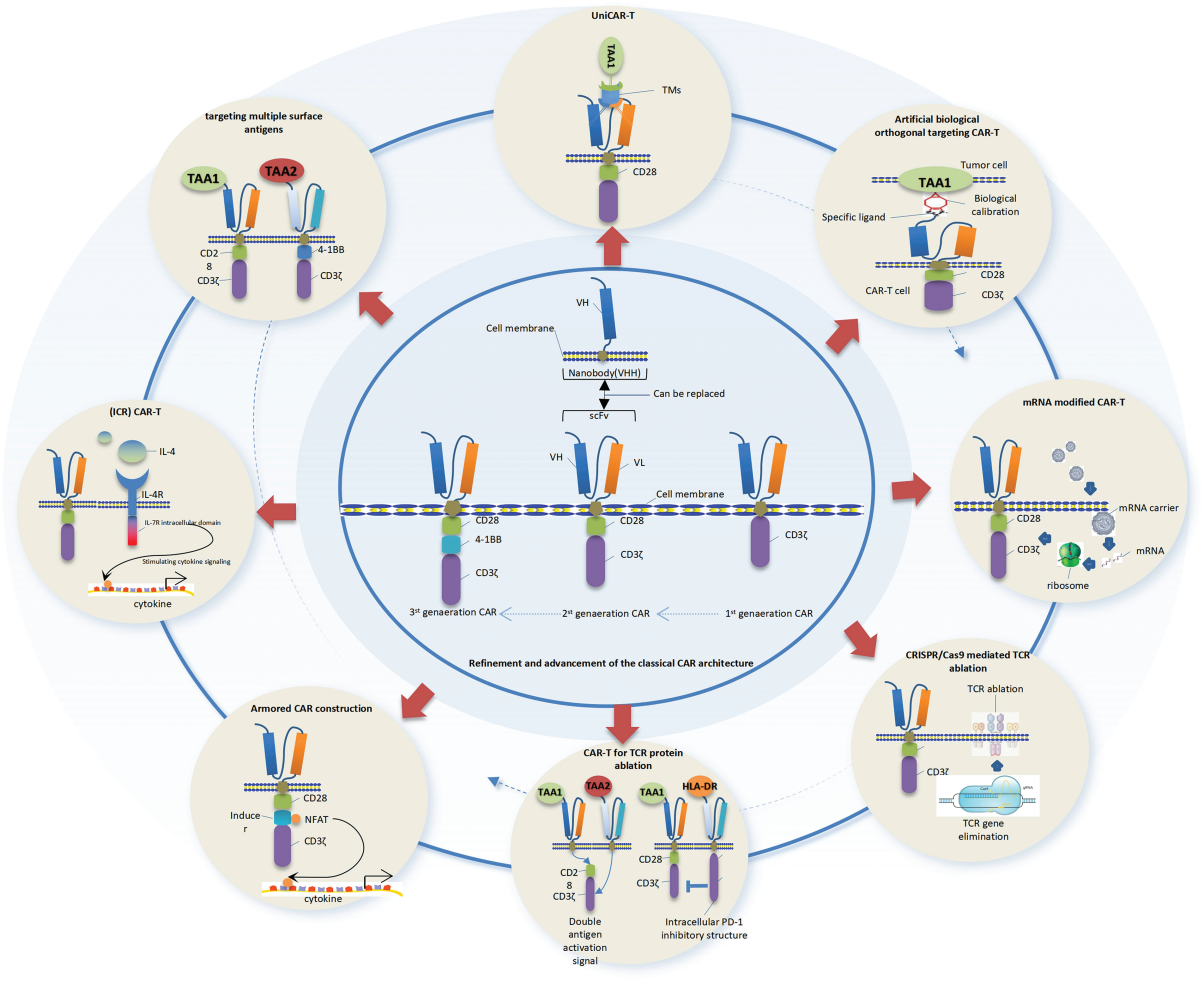
Summary diagram illustrating the developmental trajectory and ongoing optimization of CAR–T cell design. The figure portrays the evolution of structural designs from initial generation to current iterations, with varying design timelines that do not follow a sequential order. Preclinical research holds intrinsic value, but long-term examination is essential for assessing actual clinical efficacy.

The development and structural optimization of CAR–T cells are shown in Fig. 2.

### Synergies of “CAR–T” therapy in combination with other therapies

Currently, the clinical assessment of immunotherapy primarily focuses on the host’s antitumor immune response as a single-step monotherapy. However, considering the intricate immune evasion mechanisms within the tumor microenvironment, it is improbable that monotherapy alone can surmount this prominent obstacle impeding antitumor immunity in patients. Similarly, in tumor CAR–T immunotherapy, the efficacy of monotherapy is limited and additional strategies are required to optimize therapeutic outcomes. Efficient combination strategies should be implemented across four distinct treatment nodes. The nodes encompass the following aspects: elimination of immunosuppression; induction of immunogenic tumor cell death; enhancement of antigen presentation or adjuvantability; and promotion of activation and survival of memory T cells, or augmentation of macrophage effector function. In the past decade, significant efforts have been devoted to augmenting the efficacy of tumor immunotherapy by integrating CAR–T cells with other cancer therapies, particularly in the context of solid tumors. Through continuous research and practice, the advancement of CAR–T cells in conjunction with complementary therapies presents promising prospects for achieving more efficacious cancer treatment. The combination therapy yields promising outcomes in terms of tumor regression and enhanced cancer control compared to monotherapy. According to the diverse mechanisms underlying tolerance towards individual treatments, a multitude of drug combinations are employed in clinical practice. These combined therapeutic approaches encompass chemotherapy, radiotherapy, oncolytic viruses, tumor vaccines, cytokines, checkpoint inhibitors, BiTEs, immunomodulators, hematopoietic stem cell transplantation (HSCT), and other related interventions. It has the potential to overcome tumor immune evasion by modulating the tumor microenvironment, establishing a connection between CAR–T cells and tumor cells, and potentially targeting a diverse range of antigens. Meanwhile, it curtails the untoward effects of CAR–T cell therapy, thereby augmenting the clinical response. The proposed treatment strategy is characterized by its simplicity, feasibility, and promising potential, rendering it a compelling approach. A comprehensive review has been conducted to thoroughly examine the pertinent content [219]. The subsequent section presents an overview of several recent studies.

#### Reinforced by immune checkpoint blockade to ameliorate immunosuppression

Following activation induction, T cells undergo up-regulation of co-inhibitory pathways in response to repeated antigen stress, thereby constraining the efficacy of anti-tumor immune responses. However, exposure of tumor cells to Th1 cytokines secreted by T cells upregulates the expression of coinhibitory ligands, such as programmed death-ligand 1 (PD-L1). Furthermore, in adoptive CAR–T cells, depleted CAR–T cells frequently exhibit heightened expression of inhibitory checkpoint receptors such as PD-1, accompanied by an upregulation of PD-1 ligands (PD-L1 and PD-L2) on tumor cells. It can potentiate the immune checkpoint signaling pathway, thereby further suppressing the immune functionality of CAR–T cells and inducing adaptive drug resistance, consequently compromising the overall efficacy of CAR–T cells. In addition to PD-1, multiple coinhibitory checkpoint receptors including TIM-3, LAG3, and TIGIT are observed to be upregulated on dysfunctional T cells. In order to establish the functional persistence of CAR–T cells in the immunosuppressive microenvironment and achieve long-term antitumor efficacy, it is imperative to overcome this redundant mechanism of immunosuppression within the TME. Currently, the most promising approach to enhance anti-tumor therapeutic immunity involves mitigating immunosuppression through immune checkpoint blockade. The combination of CAR–T cell immunotherapy and checkpoint blockade represents a highly promising strategy for treating solid tumors. Antibody-mediated checkpoint blockade effectively reverses systemic immunosuppression, potentially leading to the activation of autologous tumor immune responses. T-cell engineering, on the other hand, enables targeted transduction of receptors that specifically counteract tumor-induced immunosuppression in designated T cells. Concomitant blockade of multiple checkpoint inhibition pathways synergistically augments the efficacy of CAR–T cells. Cherkassky et al. employed a retroviral vector to combine mSLN-CAR-mediated costimulation with a PD-1 dominant negative receptor (PD-1 DNR), which competes with the endogenous PD-1 receptor for binding to PD-1 ligands (PD-L1 and PD-L2) and competitively inhibits the PD-1/PD-L1/2 signaling pathway, thereby providing intracellular checkpoint blockade [220]. The combination of costimulation and checkpoint blockade synergistically enhanced T-cell function in the presence of PD-L1 expression, resulting in sustained tumor-free survival in animal models following a single low-dose infusion of CAR–T cells.

Due to the intricate nature, substantial expenses, and technical challenges associated with engineering T cells, it has been observed that the combination of CAR–T therapy and immune checkpoint inhibitors (such as PD-1 antibodies) can also augment the effectiveness of anti-tumor immunotherapy. The study conducted by Cherkassky et al. also demonstrated the efficacy of an enhanced CD28/CD3ζCAR–T targeting MSLN (M28z) in combination with a PD-1-targeting antibody, yielding optimal results through repeated dosing [220]. Treatment with PD-1-blocking antibodies by Chen et al. was able to partially rescue effector function of M28z *in vitro* and *in vivo* and significantly reduce tumor burden [116]. However, upon discontinuation of the antibody treatment, tumor recurrence was observed. Although long-term and repeated antibody therapy was effective in controlling tumor progression, complete eradication of the tumor was not achieved. The PD-1 blocking antibody is believed to possess the potential to restore M28z function; however, its effect exhibits transience, necessitating repeated administrations of the antibody for effective inhibition of tumor progression. Compared to M28z cells, PD-1 DNR co-transduced M28z CAR–T cells exhibited augmented proliferation, enhanced cytotoxicity, and increased cytokine secretion upon repeated antigenic stimulation. The administration of M28z PD-1 DNR cells resulted in enhanced tumor burden control and prolonged median survival in mice.

In summary, the utilization of PD-1 antibody strategy alleviates the suppression of endogenous tumor-reactive T cells, thereby facilitating the mobilization of systemic anti-tumor efficacy in immune cells, particularly for fostering bystander immune cell-mediated immune responses subsequent to the release of diverse tumor antigens resulting from tumor cell destruction. However, the PD-1 DNR strategy is utilized to augment the response of CAR–T cells that are transferred adoptively, and these two strategies possess distinct clinical applications and potential adverse reactions. The superiority of each strategy is influenced by numerous factors, yet no comprehensive evaluation report has been identified.

#### In conjunction with molecular targeted inhibitors

The AKT/mTOR pathway is frequently dysregulated in tumors and serves as a pivotal regulator of cellular growth and survival. mTOR facilitates tumorigenesis and progression by orchestrating tumor cell proliferation, angiogenesis, as well as the upregulation of genes associated with chemotherapy and immune evasion. Small-molecule inhibitors, such as rapamycin (rapa) and rapalogs, can effectively target the mTOR pathway due to their similar mechanisms of action. mTOR inhibition can elicit a spectrum of cellular effects, encompassing the downregulation of MCL-1 anti-apoptotic factors, as well as inhibitory cytokines and ligands such as interleukin-10 (IL-10),VEGF, and PD-L1. The aforementioned effects generally augment T-cell functionality and viability, while concurrently heightening the susceptibility of tumors to immune eradication. However, the potential therapeutic benefits of these agents are counteracted by their direct inhibitory effects on effector T cells. The T cells were genetically modified by Huye et al. to express a mutant form (mTorRR) of mTorS2035 that confers resistance to rapa, along with a CAR targeting CD19 expressed on B-cell malignancies (CAR.CD19-28ζ) [221]. The mTorRR mutant of this engineered cell exhibits impaired binding to rapamycin, while maintaining its overall functionality. T cells expressing mTorRRs sustain mTor signaling in the presence of rapa, enabling their expansion and functionality comparable to T cells cultured without the drug. Furthermore, the incorporation of rapa with RAPA-resistant T cells, via coexpression of CAR, confers tumor specificity. Compared to treatment alone, CAR.CD19-28ζ demonstrated superior anti-tumor activity *in vitro* against both Burkitt lymphoma and acute lymphoblastic leukemia cell lines. This was attributed to rapa’s ability to inhibit tumor cell proliferation, down-regulate the anti-apoptotic factors Mcl-1 and Bcl-xL, as well as suppress the production of inhibitory cytokines IL-10 and VEGF. Therefore, the utilization of mTorRR-modified T cells can effectively counteract the detrimental impact of rapa on effector T cells, presenting a promising combinatorial approach for cancer therapy that harnesses the immunomodulatory properties of pharmaceutical agents.

EGFR represents a promising therapeutic target for the treatment of triple-negative breast cancer (TNBC). The third-generation EGFR CAR–T cells exhibited robust and selective cytotoxicity against TNBC. However, the majority of patients fail to derive therapeutic benefits from immunotherapy due to primary drug resistance, while a subset of responders experience relapse following treatment as a result of acquired drug resistance. There is compelling evidence suggesting that epigenetic regulation can augment patients’ responsiveness to immunotherapy, implying a potential association between immune tolerance and epigenetics, which warrants further investigation [222]. Through transcriptome analysis of EGFR CAR–T cell-resistant TNBC tumors, Xia et al. discovered a substantial upregulation of genes associated with immunosuppression, suggesting that the augmented expression of numerous immunosuppressive molecules following EGFR CAR–T cell treatment constituted the primary cause for the development of resistance against CAR–T therapy [223]. Studies have demonstrated that the regulation of these immunosuppressive molecules primarily relies on IFN-γ signaling, thereby suggesting their potential activation by CAR–T cell-derived IFN-γ. The activation of CAR–T cells was found to induce a series of immunosuppressive genes, which were associated with enhancers specifically activated by CAR–T cells. Moreover, the expression of these genes could be effectively suppressed through the use of inhibitors targeting transcriptional regulators. The CDK7 inhibitor THZ1, which effectively suppresses CDK7-mediated phosphorylation of Pol II, exhibits remarkable sensitivity towards these genes. The RNA-seq analysis also demonstrated that the “Achilles cluster” of TNBC-specific genes, which are associated with super-enhancers, exhibits heightened sensitivity to CDK7 inhibition/THZ1 treatment [224]. The enhanced therapeutic efficacy of THZ1 may also be attributed to its inhibitory effect on these genes. The combined treatment of THZ1 and EGFR CAR–T cells demonstrates significant inhibition of immune resistance, tumor growth, and metastasis in TNBC xenograft and allograft models in mice based on animal experiments, thereby presenting a novel strategy for further clinical investigation.

In summary, CAR–T combination therapy encompasses the intricate interplay between immune cells, tumor cells, and the microenvironment. Among these factors, researchers primarily focus on harnessing synergistic and complementary effects to augment clinical efficacy; however, given the complexity of molecular signaling networks involved, potential antagonistic or adaptive tolerance effects cannot be disregarded. After inhibiting the AKT/mTOR pathway, although the drug-resistant mutant (mTorRR) CAR–T cells can be tolerated, bystander immune cell function remains suppressed, thereby impeding tumor cell clearance and potentially compromising therapeutic efficacy. In the context of synergistic therapeutic strategies involving epigenetics, precise regulation of downstream transcriptional regulators in the IFNγ signaling pathway assumes paramount importance due to the dual nature of IFNγ, which can act both as a cytotoxic agent and a protective factor. On one hand, it serves as an initial immune response to the activation and differentiation of T cells, thereby enhancing the functionality of effector T cells. Conversely, under certain circumstances such as chronic exposure, IFNγ has been implicated in promoting tumor progression and/or drug resistance. The precise mechanism underlying the induction of immunosuppressive genes by IFNγ exposure remains to be elucidated. Various CDK7 enhancer inhibitors have been observed to exhibit distinct effects [223]. The relationship between downstream proteins and the IFNγ-JAK-STAT pathway remains ambiguous, lacking clarity in current literature. Hence, the efficacy of these combined interventions in clinical settings necessitates validation through extensive basic and clinical investigations to overcome the barriers for their practical implementation.

### Impact of specific loci on the functionality of CAR–T cells during the integration of CAR transgenes

The integration of CAR transgenes at different loci can modulate the level of CAR expression, while the exogenous integrated DNA fragments may impact neighboring endogenous genes and chromatin structure, potentially altering the behavior and function of transduced T cells, and even facilitating cellular transformation. During CAR-based therapy, the desired CAR structure can be introduced into target immune cells through viral particles, mRNA, and transposons. Virus-based vectors, such as retroviral (RV) and lentiviral (LV) vectors, are the most commonly employed methods for achieving stable CAR gene expression in T cells due to their high efficiency of gene transfer and ability to maintain consistent CAR expression levels [225]. The vectors employed in this study facilitate the semi-random integration of CAR transgenes into diverse genomic loci, exhibiting a preference for highly expressed genes and accessible chromatin regions. The utilization of non-viral methodologies presents a cost-effective strategy for the engineering of CAR–T cells [226]. For instance, the Sleeping Beauty transposon (SB), a non-viral approach comprising the exogenous CAR gene and transposon structure, is introduced into the target cell using cationic polymers or electroporation as an efficient delivery system. In this approach, transgenes can be integrated into genomic loci distal to highly expressed genes or oncogenes [226]. However, there exist concerns regarding the safety and clinical applicability of this non-viral approach, which introduce uncertainties. The integration of a transgene may exert an influence on the expression and/or chromatin architecture of neighboring genes [227], This phenomenon may induce disruptions in the functionality of engineered effector cells, potentially driving them towards tumor transformation. One prevalent occurrence is the acquisition of dominant functional mutations [228]. The inclusion of such randomly inserted genes may be influenced by their genomic location and subjected to epigenetic silencing, thereby compromising the reliability and predictability of their expression. For instance, centromeres and proximal telomeres represent loci where exogenous genes are particularly prone to transcriptional silencing [228]. Therefore, in order to achieve the production of engineered CAR–T cells that are stable, reliable, and safe, it is imperative to carefully select appropriate integration sites. Emerging technologies, such as recombinant adeno-associated virus (rAAV), megacribonuclease, Zinc finger nuclease (ZFN), transcriptional activator-like effector nuclease (TALEN), and CRISPR-Cas9, enable targeted integration of DNA fragments into specific loci within the human genome. For instance, the precise integration of genes at specific sites through homologous recombination and adeno-associated virus (AAV), as well as the appropriate transfer and expression of CAR constructs in primary T cells, exemplify efficient techniques [229]. Where is the optimal genomic locus within T cells to integrate the CAR gene, ensuring both maximum safety and effectiveness? That is, how can the genomic safe harbor (GSH) be accurately identified? The GSH-integrated CAR transgenes function appropriately in a predictable manner, without disrupting the activity of endogenous genes or inducing carcinogenic chromosomal translocations. Regrettably, there exists a knowledge gap. Papapetrou et al. formulated the definition of GSH based on a comprehensive analysis using computer algorithms and in-depth examination, considering five distinctive criteria: 1. The distance from any given gene should be ≥50 kb. 2. The distance from any cancer-related gene should be ≥300 kb. 3. The distance from any miRNA gene should be ≥300 kb. 4. Integration events should not occur within the transcription unit. 5. Transgene integration within ultra-conserved regions of the genome is not permissible [230]. Odak and colleagues assessed an algorithm to identify exogenous GSHs (eGSHs) in human T lymphocytes, which could be utilized for CAR transgene integration to achieve sustainable CAR expression, thereby circumventing spontaneous CAR stimulation and T cell terminal differentiation [231]. Mitigate cytotoxicity associated with transgene integration by circumventing integration into functional genomic elements, averting transgene silencing, and enhancing the efficiency of CRISPR-Cas9 [231]. Non-coding RNAs (ncRNAs) have been shown to play crucial roles in a diverse range of cellular and physiological processes, including but not limited to gene expression and regulation, chromatin dynamics, differentiation, and development [232]. Disruption or dysregulation of non-coding RNAs (ncRNAs) can contribute to the development of cancer and immune disorders. Therefore, Odak introduced an additional criterion: transgenic integration should not interfere with the functionality of ncRNAs [231]. In order to achieve effective site-directed transgene integration into the genome, Odak defines the seventh characteristic criterion as follows: the corresponding nuclease must exhibit efficient accessibility and cleavage ability at the target site [231]. Odak and colleagues also introduced an eighth criterion based on chromatin structure: the transgene expression and regulation must not interfere with or inhibit other cellular processes [231]. The study conducted by Odak and colleagues demonstrated that transgenic integrated T cells, which were genetically modified to express CD19-redirected chimeric antigen receptor (CAR) known as GSH6, exhibited favorable therapeutic outcomes in preclinical mouse models while also displaying resistance to tumor re-challenges even after 100 days of administration [231]. Currently, the following three sites are widely acknowledged as target sites for CAR integration in the context of gene editing: (1) AAVS1; (2) CCR5 gene; (3) Human Rosa26 locus. Hamed Dabiri has reviewed previous studies that suggest integrating CAR coding sequences into TCR sites and placing them under the control of endogenous regulatory elements can reduce tonic signaling, prevent accelerated T cell differentiation and failure, and enhance the therapeutic efficacy of engineered CAR–T cells [233,212]. Kinetic measurements of antigen-induced CAR internalization and degradation suggest that the expression and cell surface dynamics of CAR are contingent upon enhancer/promoter elements [212]. These findings suggest that strict transcriptional regulation of CAR expression is crucial for effective tumor eradication. Therefore, integrating CAR transgenes into TCR sites can minimize the risk of insertional tumorigenesis and TCR-induced autoimmunity and allogeneic reactions, resulting in safer CAR–T cell therapy. Ultimately, more efficient CAR–T cell products can be obtained by reducing constitutive signaling and delaying T cell exhaustion.

## Discussion

The application of adoptive immunotherapy utilizing CAR–T cells presents a novel clinical approach for the treatment of neoplastic disorders. This is particularly applicable to patients diagnosed with hematological malignancies [4,5]. Significant advancements have been made in the treatment of hematologic malignancies, including B-cell acute lymphoblastic leukemia (B-ALL), multiple myeloma (MM), and non-Hodgkin lymphoma (NHL). The US Food and Drug Administration (FDA) has granted approval for CAR–T cell therapies targeting CD19 and B-cell maturation antigen (BCMA) to treat relapsed/refractory B-cell malignancies. These approved therapies include tisagenlecleucel (Kymriah), axicabtagene ciloleucel (Yescarta), ciltacabtagene autoleucel (Carvykti), idecabtagene vicleucel (Abecma), brexucabtagene autoleucel (Tecartus), and lisocabtagene maraleucel (Breyanzi). Simultaneously, a multitude of novel clinical studies pertaining to CAR–T immunotherapy have received regulatory approval from diverse countries, with numerous trials currently underway and actively recruiting participants. However, the analysis of these applied and clinical studies based on available data also sheds light on significant clinical challenges, encompassing treatment resistance observed in a subset of patients, hurdles associated with the transition to solid tumors, as well as treatment-related toxicity [6]. These adverse factors impede the clinical application of CAR–T, thereby prompting increased research efforts towards analyzing and addressing these challenges.

The development of drug resistance following CAR–T therapy is typically attributed to the rapid decline or loss of targeted tumor antigens post-treatment, leading to a subsequent loss in targeting efficacy. Under the pressure of immune editing, the original tumor cells expressing the targeted TAA are eliminated, giving rise to a subsequent emergence of new tumor cells lacking the aforementioned target antigen. Consequently, this loss of antigenic activation potential renders CAR–T cell therapy ineffective and leads to an unresponsive treatment outcome. The utilization of CAR–T therapy targeting multiple TAA targets by investigators represents a highly effective and strategic approach. Multiple cars can be engineered on a single T cell, or CAR–T cells with distinct targets can be administered sequentially based on antigen expression results, thereby mitigating the occurrence of relapse or non-response instances. This challenge arises from the inherent heterogeneity of tumors, which poses a significant impediment to targeted therapy. The implementation of a multi-target attack strategy is still significantly hindered by the high cost of treatment, technical complexity, and increased risk of adverse reactions. Therefore, an in-depth exploration of this approach is imperative to achieve optimal therapeutic efficacy. Addressing the challenge of leveraging adoptive targeted therapy-induced immune responses to enhance tumor eradication within the autoimmune system represents a pivotal concern. In principle, the destruction of tumor cells by a single target has the potential to release a plethora of tumor cell membrane or membrane-related antigens, thereby facilitating antigen presentation and subsequent activation of the systemic immune system, ultimately leading to enhanced tumor clearance. However, it is important to note that solid tumors can still be influenced by a range of immunosuppressive, physical, or chemical factors within the body due to their unique structural characteristics. The exploration of comprehensive solutions to address these challenges warrants further investigation in future research.

The landscape of CAR–T immunotherapy has undergone a significant transformation, shifting its focus from hematological malignancies to encompass the realm of solid tumors. The immunosuppressive microenvironment within solid tumors constitutes the primary impediment to achieving a favorable response in CAR–T targeted therapy. The current state of research necessitates further investigation into a diverse range of theoretical solutions. Firstly, this approach offers a relatively straightforward, feasible, and efficacious strategy to directly disrupt or eradicate the tumor microenvironment while simultaneously releasing tumor antigens in conjunction with conventional modalities such as radiotherapy, chemotherapy, and surgery. Subsequently, when combined with immunotherapeutic techniques like CAR–T cell therapy, it maximizes the potential of targeted immunity and bystander cellular immunity to enhance patients’ response rates. Furthermore, gene modification technology can be employed to induce the expression of specific immune cytokines, chemokines, or inhibitory receptors in CAR–T cells, thereby counteracting detrimental factors within the tumor microenvironment. This strategy enhances the survival, proliferation, and differentiation capacity of CAR–T cells while augmenting their cytotoxic efficacy. In terms of adverse reactions, the “on-target off-tumor effect” arises due to the absence of tumor-specific antigens. Physiological levels of TAA are typically expressed in healthy tissues. Excessive CAR avidity does not elicit a more potent immune clearance response against tumors; instead, it may exacerbate toxic effects on healthy tissues, such as neurotoxicity. The quantitative disparity in TAA expression between tumor and normal tissues can be utilized to establish an appropriate CAR avidity threshold, thereby reducing the probability of CAR–T cells interacting with normal tissues and minimizing the ‘on-target off-tumor effect’. Alternatively, the surface of tumor cells can be calibrated with specific molecules, and CAR can utilize paired specific receptors to target tumor cell markers specifically, thereby achieving the capability of complete tumor elimination without causing damage to normal tissue cells. Simultaneously, the suicidal or regulatory mechanisms of helper CAR cells can be employed to timely terminate the functionality of CAR–T cells in case of severe adverse reactions and mitigate irreversible hazardous consequences. The implementation of these multiple safety measures has significantly enhanced the safety profile of CAR–T therapy.

Due to the unique nature of adoptive cell therapy product manufacturing, the quality of CAR–T cells directly impacts their ultimate clinical application. The implementation of a large-scale GMP protocol holds significant importance in facilitating mass production applications. However, due to the intricate production technology and process involved in CAR–T cell manufacturing, it is not feasible to establish a universally applicable GMP protocol for CAR–T cells across different tumor types. By enhancing standardized operational procedures based on diverse production purposes and process steps, optimizing the utilization of existing fully automated large-scale cultivation production equipment, identifying appropriate process parameters, minimizing human intervention, establishing a manufacturer’s quality management system, and constructing targeted product GMP agreements, the stability of product quality can be effectively maintained while eliminating uncertainties caused by influencing factors. The production cost of CAR–T products is high, and the clinical benefit population for promotion is limited. The reduction of costs is also a crucial factor in overcoming the barriers to its clinical implementation. The utilization of adapter modules or bispecific antibody adapter structures in universal CAR–T products presents a viable strategy, necessitating further investigation into their clinical advantages.

In summary, the field of CAR–T immunotherapy is influenced by numerous factors. With the comprehensive investigation of TME and understanding of tumor immune mechanisms, it is anticipated that additional challenges will be identified and elucidated, leading to the exploration of more effective strategies. This iterative progression will inevitably pave the way for a groundbreaking advancement in tumor immunotherapy.

## Data Availability

All information or data in this review are from publicly published international journals.
